# Implementation of Patient-Reported Outcome Measures for Gender-Affirming Care Worldwide: A Systematic Review

**DOI:** 10.1001/jamanetworkopen.2023.6425

**Published:** 2023-04-03

**Authors:** Rakhshan Kamran, Liam Jackman, Cynthia Chan, Yujin Suk, Chloe Jacklin, Eve Deck, Nina Wietek, Melissa Stepney, Conrad Harrison, Abhilash Jain, Jeremy Rodrigues

**Affiliations:** 1 Nuffield Department of Orthopaedics, Rheumatology and Musculoskeletal Sciences, University of Oxford, Oxford, United Kingdom; 2 Temerty Faculty of Medicine, University of Toronto, Toronto, Ontario, Canada; 3 Department of Family Medicine, University of Ottawa, Ottawa, Ontario, Canada; 4 Department of Biochemistry and Biomedical Sciences, McMaster University, Hamilton, Ontario, Canada; 5 University of Oxford, Oxford, United Kingdom; 6 Department of Family Medicine, Western University, London, Ontario, Canada; 7 Nuffield Department of Women’s and Reproductive Health, University of Oxford, Oxford, United Kingdom; 8 Centre for Academic Primary Care, University of Bristol, Bristol, United Kingdom; 9 Warwick Clinical Trials Unit, University of Warwick, Coventry, United Kingdom; 10 Department of Plastic Surgery, Stoke Mandeville Hospital, Buckinghamshire Healthcare NHS Trust, Aylesbury, United Kingdom

## Abstract

**Importance:**

Gender-affirming care is a key clinical area that can benefit from implementation of patient-reported outcome measures (PROMs). Identifying barriers to and enablers of PROM implementation is needed to develop an evidence-based implementation strategy.

**Objective:**

To identify (1) PROMs previously implemented for gender-affirming care and constructs measured, (2) how patients completed PROMs and how results were reported and used, and (3) barriers to and enablers of PROM implementation.

**Evidence Review:**

In this systematic review, PubMed, Embase, MEDLINE, PsycINFO, CINAHL, and Web of Science were searched from inception to October 25, 2021, and updated on December 16, 2022. Gray literature was searched through gray literature database, online search engine, and targeted website searching. Inclusion criteria were (1) original articles of (2) a formally developed PROM or ad hoc instrument administered for gender-affirming care to (3) patients accessing gender-affirming care. The Critical Appraisal Skills Programme tool was used to evaluate quality of included studies. This review was registered on PROSPERO (CRD42021233080).

**Findings:**

In total, 286 studies were included, representing 85 395 transgender and nonbinary patients from more than 30 countries. A total of 205 different PROMs were used in gender-affirming care. No studies described using an implementation science theory, model, or framework to support PROM deployment. Key barriers to PROM implementation included issues with evidence strength and quality of the PROM, engaging participants, and PROM complexity. Key enablers of PROM implementation included using PROMs validated for gender-affirming care, implementing PROMs able to be deployed online or in person, implementing PROMs that are shorter and reduce patient burden, engaging key stakeholders and participants as part of developing an implementation plan, and organizational climate.

**Conclusions and Relevance:**

In this systematic review of barriers to and enablers of PROM implementation in gender-affirming care, PROM implementation was inconsistent and did not follow evidence-based approaches in implementation science. There was also a lack of patient input in creating implementation strategies, suggesting a need for patient-centered approaches to PROM implementation. Frameworks created from these results can be used to develop evidence-based PROM implementation initiatives for gender-affirming care and have potential generalizability for other clinical areas interested in implementing PROMs.

## Key Points

**Question:**

What are the barriers to and enablers of implementing patient-reported outcome measures (PROMs) in gender-affirming care?

**Findings:**

In this systematic review of 286 studies, the main barriers to implementing PROMs in gender-affirming care were issues with evidence strength and quality of the PROM, engaging participants, and complexity of the PROM.

**Meaning:**

This study’s findings may help to guide PROM implementation initiatives for gender-affirming care with potential generalizability to other clinical areas.

## Teaser

This systematic review identifies patient-reported outcome measures for gender-affirming care and the barriers to and enablers of implementation.

## Introduction

Patient-reported outcome measures (PROMs) are self-report instruments used to measure elements of health from patients’ own perspectives.^1^ These measures can enable comparisons of clinicians’ performance, driving service improvements.^2^ By helping to identify bothersome symptoms, PROMs can enhance communication between clinicians and patients,^3^ enhance patient satisfaction,^4^ and improve care outcomes,^5^ including mortality.^6^ However, recent evidence suggests that the benefits of PROMs are lost without evidence-based implementation strategies to ensure they gain traction and uptake.^7,8^

One area that may benefit from widespread and organized PROM implementation is gender-affirming care. Gender-affirming care comprises a range of psychosocial, hormonal, and surgical care offered to transgender and nonbinary individuals to help with a person’s gender transition. However, not all gender identity service delivery is gender affirming due to pathologization, discrimination, and cisnormativity existing in some gender identity centers.^9^ Clinical guidance and international standards emphasize that individual patient needs must be comprehensively understood to offer gender-affirming care in line with patient goals.^10^ Implementation of PROMs can improve gender-affirming care through regular monitoring of patient satisfaction (through patient-reported experience measures) and facilitate studies of treatment effectiveness and cost-effectiveness, supporting access to gender-affirming care.^11,12^ Well-conducted implementation of relevant and high-quality PROMs may also provide evidence and measurement of quality and standard of care received by patients; drive patient-centered care through improving communication between patients and clinicians; guide shared decision-making and facilitate open dialogue between clinicians and patients, challenging bias where appropriate; and inform service development and improvement. To develop an implementation plan for PROMs in gender-affirming care, barriers and enablers to implementation must be understood, and an evidence-based PROM implementation plan is needed.^13,14,15^

Implementation science offers approaches to identify implementation barriers and enablers, develop an implementation strategy, and evaluate implementation effectiveness.^7,13,16^ Normalization process theory is particularly applicable for PROM implementation and describes how innovations become routinized.^13,17^ The Knowledge to Action model applies to PROM implementation through describing steps to translate research into practice.^13,18^ The Consolidated Framework for Implementation Research (CFIR) can be used to systematically categorize barriers to and enablers of implementation and is known as a meta-framework that incorporates key concepts from implementation science into 1 framework.^19^ The CFIR also allows for systematic linking of identified barriers to implementation strategies and has been applied successfully in PROM implementation.^16,19^ The aims of this systematic review are to (1) identify PROMs previously implemented for use in gender-affirming care and identify constructs measured; (2) identify how patients complete PROMs and how results are reported and used; and (3) identify barriers to and enablers of PROM implementation for use in gender-affirming care, categorizing results using the CFIR. We use the results of this systematic review to create a framework that can be used to develop PROM implementation initiatives for gender-affirming care.

## Methods

This systematic review followed the Preferred Reporting Items for Systematic Reviews and Meta-Analysis reporting guideline^20^ and was registered with the International Prospective Register of Systematic Reviews (CRD42021233080). This study was reviewed by the Clinical Trials and Research Governance Body at the University of Oxford, who determined that this study did not require ethics approval, as this review did not collect human participant data.

### Patient and Public Involvement

Six patient and public partners were involved in designing and conducting this review, including confirming the relevance of the research question, aiding with search strategy development (identifying key terms and gray literature sources), and confirming applicability of findings. Patient and public partners represented individuals from the transgender and nonbinary community recruited through representatives from national transgender charity organizations and community support groups.

### Eligibility Criteria

We included original articles evaluating a formally developed PROM (a self-report tool created from mixed-methods PROM development or validation studies) or ad hoc instrument (a self-report tool that has not undergone formal development or validation). The PROM had to be administered to patients accessing gender-affirming care (psychosocial, hormonal, and surgical care offered to transgender and nonbinary individuals to help with a person’s gender transition) or where subgrouped data were available for patients accessing gender-affirming care.

### Search Strategy and Selection Process

Six databases, including PubMed, Embase, MEDLINE, PsycINFO, CINAHL, and Web of Science, were searched from inception to October 25, 2021. The search was updated on December 15, 2022. There were no restrictions on language, country of origin, or publication date. Gray literature was searched through gray literature databases (eg, OpenGrey), customized searching via online search engine, and targeted searching of relevant websites (eAppendix 1 in Supplement 1). The search strategy was developed with patient and public partners, an information scientist, and an implementation scientist outside of the author group.

Articles underwent title and abstract screening and subsequent full-text screening by 2 reviewers independently, using the Covidence review management platform (R.K., L.J., C.C., Y.S., C.J., E.D., N.W.), with conflicts resolved by a third reviewer. Two reviewers (R.K., L.J., C.C., Y.S., C.J., E.D., N.W.) extracted data from the included articles independently, with conflicts resolved by a third reviewer. Non-English articles were screened and extracted by a reviewer (L.J., N.W.) fluent in the article’s original language and a second reviewer (R.K.) using artificial intelligence–powered translation software (DeepL Translator; DeepL).

### Data Items

Extracted data included study information (country, clinic setting, study design, implementation theory, and level of evidence), PROM characteristics (instrument used; administration purpose; constructs measured; number of items; timing, location, mode, and frequency of administration; completion rate; data security; training for staff; how results were displayed and used; budget considerations; and data collection platform used), and barriers to and enablers of PROM implementation categorized according to CFIR constructs. Data items were chosen based on key concepts in PROM implementation and CFIR.

### Risk-of-Bias Assessment and Synthesis Methods

The Critical Appraisal Skills Programme tool^21^ was used by 2 independent reviewers (R.K., L.J., C.C., Y.S., C.J., E.D., N.W.) to evaluate the quality of included studies, with conflicts resolved by a third reviewer. The CFIR guidance was followed to categorize barriers to and enablers of PROM implementation by 2 independent reviewers (R.K., L.J.).^19^ Patient and public partners reviewed CFIR-based frameworks generated from this study to confirm their relevance and applicability.

### Statistical Analysis

Descriptive statistics were used to report demographic characteristics and study information. Agreement for CFIR categorization was calculated through identifying the percentage of overlap between reviewers. Discrepancies with CFIR categorization were resolved through discussion between reviewers (R.K., L.J.). Analyses were performed using Microsoft Excel, version 2019 (Microsoft Corporation) software. Guidance from Synthesis Without Meta-Analysis^22^ was followed for data synthesis reporting.

## Results

### Study Selection and Characteristics

A total of 17 380 records were identified from the search, with 286 studies^23,24,25,26,27,28,29,30,31,32,33,34,35,36,37,38,39,40,41,42,43,44,45,46,47,48,49,50,51,52,53,54,55,56,57,58,59,60,61,62,63,64,65,66,67,68,69,70,71,72,73,74,75,76,77,78,79,80,81,82,83,84,85,86,87,88,89,90,91,92,93,94,95,96,97,98,99,100,101,102,103,104,105,106,107,108,109,110,111,112,113,114,115,116,117,118,119,120,121,122,123,124,125,126,127,128,129,130,131,132,133,134,135,136,137,138,139,140,141,142,143,144,145,146,147,148,149,150,151,152,153,154,155,156,157,158,159,160,161,162,163,164,165,166,167,168,169,170,171,172,173,174,175,176,177,178,179,180,181,182,183,184,185,186,187,188,189,190,191,192,193,194,195,196,197,198,199,200,201,202,203,204,205,206,207,208,209,210,211,212,213,214,215,216,217,218,219,220,221,222,223,224,225,226,227,228,229,230,231,232,233,234,235,236,237,238,239,240,241,242,243,244,245,246,247,248,249,250,251,252,253,254,255,256,257,258,259,260,261,262,263,264,265,266,267,268,269,270,271,272,273,274,275,276,277,278,279,280,281,282,283,284,285,286,287,288,289,290,291,292,293,294,295,296,297,298,299,300,301,302,303,304,305,306,307,308^ included in the review (Figure). All included studies are listed in eAppendix 2 in Supplement 1. A total of 85 395 transgender and nonbinary individuals from more than 30 countries (eTable 1 in Supplement 1) were represented across the included studies. The majority of studies were conducted in the US (72; 25%), Germany (29; 10%), the Netherlands (28; 10%), Italy (18; 6%), and the United Kingdom (12; 4%). eTable 2 in Supplement 1 provides an overview of the level of evidence of the included studies. Most studies (190; 66%) were rated as level 2c, outcomes research.

**Figure.**
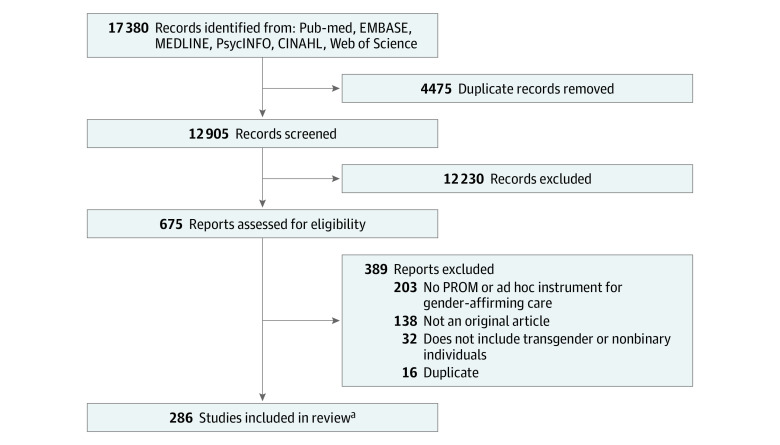
Preferred Reporting Items for Systematic Reviews and Meta-Analysis Diagram of Study Selection PROM indicates patient-reported outcome measure. ^a^Reference citations for included studies are available in eAppendix 2 in Supplement 1.

### PROMs for Gender-Affirming Care

Of the 286 included studies, 81 (28%) measured outcomes with only an ad hoc instrument. Of the remaining 205 studies, 34 (17%) used a combination of ad hoc instruments and PROMs, or PROMs only (171, 83%). Studies administered between 1 and 11 PROMs (median, 1 PROM) (Table 1).

**Table 1.** Number of Patient-Reported Outcome Measures (PROMs) Used in Included Articles and Constructs Measured by PROMs Used in Gender-Affirming Care

PROMs used and constructs measured	Frequency, No. (%)
No. of PROMs/ad hoc instruments used per study (n = 286)	
1	144 (50)
2	64 (22)
3	36 (13)
4	18 (6)
5	10 (3)
6	8 (3)
7	2 (1)
8	0
9	1 (1)
10	0
11	3 (1)
Constructs measured by PROMs (n = 205)	
Psychological functioning and mental health	77 (38)
Quality of life	26 (13)
Appearance and body image	22 (11)
Social function	20 (10)
Physical function	18 (9)
Gender-related concepts (ie, gender euphoria)	13 (6)
Voice function	12 (6)
Sexual function	11 (5)
Satisfaction with care	6 (2)

In total, 205 different PROMs were administered for gender-affirming care, varying between 1 and 250 items each (median, 20 items; mean, 26 items). The total items administered per study ranged from 1 to 284 (mean, 64 items). The PROMs covered a range of constructs, and most commonly measured psychological functioning, quality of life, and appearance (Table 1). Few PROMs measured gender-related concepts (ie, gender dysphoria and euphoria) or focused solely on satisfaction with care.

A total of 24 studies (8%) focused on pediatric populations, and 262 (92%) focused on adult populations. Patient burden was higher in pediatric gender-affirming care, with a mean of 116 items (range, 12-331 items) administered to pediatric patients vs a mean of 57 items (range, 1 to 384 items) for adult gender-affirming care.

Most studies did not report staff training on PROM administration (284; 99%). Most participants completed PROMs in the clinic (115; 40%) (Table 2).

**Table 2.** Location, Purpose, Frequency, and Mode of PROM Administration in Gender-Affirming Care Among 286 Studies

	Frequency, No. (%)
**Location of PROM completion: Where did participants complete PROMs? (n = 286)**	
In the clinic	115 (40)
At home	85 (30)
In the clinic and at home	20 (7)
Not reported	66 (23)
**Purpose of PROM implementation: What was the purpose of implementing PROMs? (n = 286)**	
Research	274 (96)
Day-to-day use	5 (2)
Both	4 (1)
Unclear	3 (1)
**Frequency of PROM administration: How many times were PROMs administered? (n = 286)**	
1	192 (67)
2	56 (20)
3	12 (4)
4	7 (2)
5	1 (1)
6	2 (1)
8	1 (1)
9	1 (1)
Not reported	14 (5)
**Mode of PROM administration**	
What platform was used to deliver PROMs? (n = 286)	
Not reported	153 (53)
Online	54 (19)
Pen and paper	31 (11)
Pen and paper by mail	30 (10)
Online or pen and paper	9 (3)
Telephone	8 (3)
Online or telephone	1 (1)

Abbreviation: PROM, patient-reported outcome measure.

Reasons for implementing PROMs varied. Most studies implemented PROMs for research use (274; 96%) (Table 2). Cost considerations were often not reported (281; 98%). No studies described the use of an implementation science theory to support PROM implementation.

Patient-reported outcome measures were administered between 1 and 9 times (mean, 2 times) (Table 2). Of the 192 studies (67%) administering a PROM only once, 111 (58%) administered the PROM as part of a baseline assessment only. A total of 81 studies (42%) administered the PROM as part of posttreatment assessment only. The remaining studies administering PROMs more than 1 time had varied follow-ups.

The mode of administering PROMs varied (Table 2). Most were administered online (54; 19%), by pen and paper in the clinic (31; 11%), or by pen and paper by mail (30; 10%). Most studies (258; 90%) did not report the data collection platform (eTable 3 in Supplement 1). Nearly all studies did not report on data security provisions (278; 97%) (eTable 4 in Supplement 1).

Scores from PROMs were most often reported as a table of means and SDs (162; 57%) (eTable 5 in Supplement 1). Results were often used to show treatment outcomes (187; 65%) or satisfaction with care (71; 25%) (eTable 6 in Supplement 1).

### Overview of Barriers to and Enablers of PROM Implementation for Gender-Affirming Care

A total of 299 codes were developed independently and in duplicate based on CFIR constructs on barriers to and enablers of PROM implementation for gender-affirming care. These codes were used to create CFIR-based frameworks that can be used to guide PROM implementation initiatives for gender-affirming care. Of the 299 codes, 282 (94%) were consistent between the 2 reviewers (R.K., L.J.). There were disagreements for 17 codes (6%), which were resolved through discussion.

#### Barriers to PROM Implementation for Gender-Affirming Care

The top 3 reported barriers to PROM implementation from the organizational perspective included issues with PROM evidence strength and quality, engaging participants, and PROM complexity. Regarding the construct of evidence strength and quality (86 codes), high variability of available PROMs used in gender-affirming care, lack of PROMs validated for gender-affirming care, and administration of PROMs for too short a follow-up time were barriers to implementation. These barriers limited the ability to incorporate results into clinical decision-making.

It was frequently mentioned in 81 codes that implementation of PROMs did not serve particular populations well; for example, patients from ethnic minority groups and those with lower socioeconomic status, lower education, and residence in rural areas were not engaged to complete PROMs. Difficulties engaging with participants after their transition were also reported due to some participants changing contact information and moving. Complexity of the PROM, specifically logistics of how to administer and score PROMs in the clinic, and online survey software failing to send out links to complete PROMs were additional key barriers mentioned in 22 codes.

The top reported barriers to PROM implementation from the patient perspective included issues with patient engagement for PROM completion and PROM complexity. Specifically, PROM length limited implementation due to higher patient burden. Furthermore, PROMs viewing gender as binary was a key barrier to implementation from the patient perspective. Table 3 provides an overview of the CFIR-based framework generated from this study on barriers to PROM implementation.

**Table 3.** Barriers to Patient-Reported Outcome Measure (PROM) Implementation in Gender-Affirming Care^a^

CFIR domain	Frequency of mention, No. of codes	Examples
**Innovation characteristics**		
Evidence strength and quality	86	High variability in PROMs, limiting standardization of implementation; lack of valid gender-specific PROMs to implement; short follow-up time that PROMs are completed, limited incorporation of results in clinical decision-making
Relative advantage	4	Lack of gender clinics using PROMs, limiting ability to compare treatment outcomes; attitudes against implementing PROMs, as benefits cannot be fully realized
Adaptability	1	PROMs limited compared with interviews or open-ended questions; issues adapting paper PROM to online display
Complexity	22	PROMs too lengthy, complex, and confusing to interpret; issues with needing to complete PROMs in person; difficulty coordinating clinic logistics of PROM administration; online PROMs unable to reach people living in rural areas, older adults, people with low socioeconomic status, and people without secure housing; paper PROMs not returned due to length; REDCap software (Vanderbilt University) failing to send survey requests; difficulty with PROM scoring; difficulty linking PROMs to the start of treatment
**Inner setting**		
Culture	1	Participants uncomfortable with answering sensitive questions due to past experiences of discrimination in health care settings
Implementation climate		
Compatibility	2	Lack of PROMs that use outdated terminology being updated to reflect current standards
**Characteristics of individuals**		
Knowledge and beliefs about the innovation	6	Belief that Likert-based PROMs are unlikely to completely capture the emotional and social complexities associated with gender transition; belief that PROMs view gender as binary
Individual identification with organization	2	Participants reluctant to visit the hospital due to association of life pretransition; participants wanting information about their transition to be private; participants afraid of the disclosure of their former identity if they completed PROMs
**Process**		
Engaging		
Innovation participants	81	Issues with engaging participants; difficulty in engaging non-White participants and those who are older, of a lower socioeconomic status, living in rural areas, and who have not completed higher education; high PROM missingness with longer-term follow-up; issues with contacting participants after treatment as some may move to a different city and change their contact information after transition; difficulty engaging with nonbinary participants
Executing	1	Lack of standardization for data collection impacts interpretability of results

Abbreviation: CFIR, Consolidated Framework for Implementation Research.

^a^ Studies are listed in eAppendix 2 of Supplement 1.

#### Enablers of PROM Implementation for Gender-Affirming Care

The top enablers of PROM implementation from the organizational perspective included evidence strength and quality, adaptability, complexity, organization needs, organization climate, and engaging key stakeholders and participants (ie, patients). Clinics found it easier to implement a PROM that demonstrated strong psychometric properties and content validity (11 codes). Using a PROM validated for gender-affirming care increased clinic staff motivation to use the PROM (8 codes). Being able to adapt PROM administration (options for pen and paper or online) and using shorter and easy-to-score instruments enabled implementation (12 codes).

Implementation success increased when PROMs were integrated into existing quality improvement initiatives and when organizations viewed PROMs as a key component of clinic appointments (8 codes). An organizational climate of wanting to improve patient monitoring and experiences and comparing outcomes between treatments enabled PROM implementation.

Also enabling PROM implementation was clinics working in partnership with key stakeholders, such as local transgender and nonbinary organizations and community members, on developing a plan for PROM administration (3 codes). Some clinics worked in partnership with local transgender and nonbinary community members who formed a stakeholder advisory group and were involved in creating a strategy for PROM implementation, which enabled PROM engagement (2 codes).

From the patient perspective, implementation was enabled with clear communication on how PROM data would be handled (8 codes), including anonymity of responses when used in research or confidentiality when used in clinical practice. Table 4 displays the CFIR-based framework generated from this study on enablers of PROM implementation.

**Table 4.** Enablers of Patient-Reported Outcome Measure (PROM) Implementation in Gender-Affirming Care^a^

CFIR domain	Frequency of mention, No. of codes	Example
**Innovation characteristics**		
Evidence strength and quality	11	Clinics keen to implement PROMs that demonstrate good psychometric properties and content validity
Adaptability	8	Increased success with implementation when patients indicate preference on mode of PROM administration; online surveys helpful in reaching patients unable to visit clinics in person
Complexity	12	Increased use of telehealth services has increased comfort with administering online PROMs; higher implementation success with shorter, simpler, and easier-to-score PROMs
**Outer setting**		
Needs and resources of those served by the organization	8	Higher implementation success when organization prioritizes measuring patient satisfaction and patient voice; regular quality improvement initiatives allow for PROM integration; implementation success when organization views PROMs as helpful for the initial clinic appointment and aiding treatment decision-making
**Inner setting**		
Networks and communications	3	Personal communication between physician and patient improves PROM implementation; patients handed PROM on arrival by receptionist and asked to complete before leaving the waiting room; receptionists explaining that data security of PROM responses aid implementation success
Culture	2	Culture of regular PROM use in the clinic improves implementation success of a new PROM
Implementation climate		
Tension for change	9	Climate in organization to better understand patient experiences, improve patient monitoring, and compare treatments enables PROM implementation; transgender advisory board for gender clinic recommending PROM use enables implementation
Compatibility	15	Climate of PROMs being crucial to the medical and surgical discipline enables implementation
Relative priority	7	Belief that measuring outcomes in gender-affirming care must be a priority enables PROM implementation
Readiness for implementation		
Leadership engagement	1	Clinic staff engaged in wanting to use PROM data to improve patient monitoring enables implementation
**Characteristics of individuals**		
Knowledge and beliefs about the innovation	3	Belief that not having PROM data limits measuring the impact of gender-affirming care
**Process**		
Engaging		
External change agents	2	Transgender advisory board recommending PROMs; transgender community leaders involved with PROM administration design
Key stakeholders	3	Involving transgender community members on planning PROM administration enables implementation
Innovation participants	8	Ensuring data security and providing participants with a transgender resource guide enables implementation success

Abbreviation: CFIR, Consolidated Framework for Implementation Research.

^a^ Studies are listed in eAppendix 2 of Supplement 1.

### Risk of Bias

Critical Appraisal Skills Programme checklists were used to assess quality of studies. In general, most studies (275; 96%) demonstrated acceptable recruitment. However, most studies (281; 99%) did not identify or include all confounding factors in their analyses. Most studies also had limited follow-up (193; 68%) (eTable 7 in Supplement 1).

## Discussion

This systematic review identifies key areas of focus relevant to PROM implementation for gender-affirming care. The CFIR-based frameworks generated from this study can be used for future PROM implementation initiatives for gender-affirming care, with potential generalizability to other clinical areas interested in implementing PROMs. The frameworks generated from our study are also living models upon which subsequent studies can build.

Overall, PROM implementation in gender-affirming care was inconsistent and did not follow evidence-based approaches in implementation science. There was a lack of patient input in creating implementation strategies for PROMs, suggesting a need for patient-centered approaches to PROM implementation in gender-affirming care. Most of the PROMs were also implemented for research purposes, which may explain why most were administered only once (eTable 5 in Supplement 1) with insufficient follow-up time. This finding suggests a need for future PROM implementation research in gender-affirming care to focus on day-to-day clinical administration.

Key barriers to PROM implementation in gender-affirming care from the patient perspective include patient engagement to complete PROMs and PROM length and complexity. Practical considerations for overcoming these barriers include communication between clinicians and patients on how PROM results will be used for research and care purposes and providing patients information on how their data will be handled securely. Due to discrimination experienced by patients in some specialist centers delivering gender-affirming care,^9^ patient and public partners emphasized confidentiality as key for PROM sections that evaluate satisfaction with health care professionals so that their care is not negatively impacted. Furthermore, applying techniques in computerized adaptive testing, a form of artificial intelligence that can reduce PROM length, may reduce patient burden of completing lengthy PROMs, resulting in higher engagement.^309^ Patient and public partners from this study also emphasized the importance of implementing a PROM in gender-affirming care that does not view gender as binary.

Key barriers to PROM implementation for gender-affirming care from the organizational perspective include complexity of administering and scoring PROMs and using PROMs not validated for gender-affirming care. Practical considerations for overcoming these barriers include selecting a PROM that has been validated for gender-affirming care, such as the Gender Congruence and Life Satisfaction Scale,^23^ the Trans Woman Voice Questionnaire,^310^ or the iTransQoL.^311^ Such consideration would ensure that the PROM has relevance for transgender and nonbinary patients and higher-quality measurement. Automating administration and scoring of PROMs may enable implementation through reducing complexity. Clinics aiming to develop PROM implementation initiatives may find increased patient engagement when working with members of the transgender community to develop an implementation plan. Aligning PROMs as an important and accepted part of clinic culture may also enable implementation success.

High variation in PROMs used and constructs measured and lack of reporting on data security for PROMs were identified. There is also a need to improve standardization of concepts measured in gender-affirming care, including consensus on the most important concepts to measure. Patient and public partners from our study emphasized the importance of data security as a key concept relevant to implementation. Information on how PROM data are stored, who can access data, and whether there is a risk that PROM data could adversely impact access or quality of care were their key concerns. Past literature is limited in reporting information on data security, representing an important future area of research for PROM implementation.

Past systematic reviews on PROMs for gender-affirming care focused on measurement properties of PROMs.^312,313^ This review is the first to focus on implementation using the CFIR. It is necessary to identify and categorize barriers and enablers to PROM implementation prior to developing PROM implementation plans, as PROM implementation strategies that are not evidence based may result in poor PROM adoption, lack of realization of PROM benefits, and research waste.^8,314^

### Strengths and Limitations

Strengths of this study include a comprehensive review of available evidence for PROM implementation in gender-affirming care and using established approaches in implementation science for data analysis. In addition, our research was conducted with patient and public partners, an essential component of PROM implementation research. Our patient and public partners confirmed face validity of the CFIR-based frameworks generated from this study.

This study has several limitations. First, there is a risk of publication bias, as this review is of published studies. Second, despite the comprehensive literature search, some articles might have been missed. Third, barriers to and enablers of PROM implementation reported in the literature focused primarily on the organizational and clinician perspectives, lacking patient perspectives. Fourth, there may have been PROM implementation attempts in gender-affirming care that were not studied or published and, thus, not covered in this review. Fifth, reasons for limited engagement among patients from ethnic minority groups and those with lower socioeconomic status, lower education, and residence in rural areas were not explored in the literature.

To overcome these limitations, our team is currently conducting a qualitative study on barriers to and enablers of PROM implementation in gender-affirming care to generate research from the patient perspective. Results from focus groups will build on the CFIR-based frameworks generated from this study, which can be used to guide PROM implementation initiatives in gender-affirming care.

## Conclusions

In this systematic review of barriers to and enablers of PROM implementation in gender-affirming care, we described key concepts relevant for evidence-based PROM implementation in gender-affirming care. These findings can be used to help to guide PROM implementation initiatives in gender-affirming care.

## Notes

Supplement 1. **eAppendix 1.** Search Strategy **eTable 1.** Overview of Countries for Articles Included in Systematic Review **eTable 2.** Oxford Centre for Evidence-Based Medicine Levels of Evidence for Included Studies **eTable 3.** Data Collection Platform for Patient-Reported Outcome Measure (PROM) Administration in Gender-Affirming Care **eTable 4.** Data Security for Patient-Reported Outcome Measures (PROMs) in Gender-Affirming Care **eTable 5.** Patient-Reported Outcome Measure (PROM) Score Reporting in Gender-Affirming Care **eTable 6.** Patient-Reported Outcome Measure (PROM) Result Application in Gender-Affirming Care **eTable 7.** Critical Appraisal Skills Programme (CASP) Checklist Results for Included Articles **eAppendix 2.** List of Included Articles

Supplement 2.
Data Sharing Statement
