# Supplementary material for: Implementation of Patient-Reported Outcome Measures for Gender-Affirming Care Worldwide: A Systematic Review
Source: JAMA Netw Open. 2023 Apr 3;6(4):e236425. doi: 10.1001/jamanetworkopen.2023.6425 (PMC10071345; doi:10.1001/jamanetworkopen.2023.6425)
Supplement: Supplement 1. — eAppendix 1. Search Strategy eTable 1. Overview of Countries for Articles Included in Systematic Review eTable 2. Oxford Centre for Evidence-Based Medicine Levels of Evidence for Included Studies eTable 3. Data Collection Platform for Patient-Reported Outcome Measure (PROM) Administration in Gender-Affirming Care eTable 4. Data Security for Patient-Reported Outcome Measures (PROMs) in Gender-Affirming Care eTable 5. Patient-Reported Outcome Measure (PROM) Score Reporting in Gender-Affirming Care eTable 6. Patient-Reported Outcome Measure (PROM) Result Application in Gender-Affirming Care eTable 7. Critical Appraisal Skills Programme (CASP) Checklist Results for Included Articles eAppendix 2. List of Included Articles [file jamanetwopen-e236425-s001.pdf]

# Supplemental Online Content

Kamran R, Jackman L, Chan C et al. Implementation of patient-reported outcome measures for gender-affirming care worldwide: a systematic review. *JAMA Netw Open*. 2023;6(4):e236425. doi:10.1001/jamanetworkopen.2023.6425

**eAppendix 1.** Search Strategy

**eTable 1.** Overview of Countries for Articles Included in Systematic Review

**eTable 2.** Oxford Centre for Evidence-Based Medicine Levels of Evidence for Included Studies

**eTable 3.** Data Collection Platform for Patient-Reported Outcome Measure (PROM) Administration in Gender-Affirming Care

**eTable 4.** Data Security for Patient-Reported Outcome Measures (PROMs) in Gender-Affirming Care

**eTable 5.** Patient-Reported Outcome Measure (PROM) Score Reporting in Gender-Affirming Care

**eTable 6.** Patient-Reported Outcome Measure (PROM) Result Application in Gender-Affirming Care

**eTable 7.** Critical Appraisal Skills Programme (CASP) Checklist Results for Included Articles

**eAppendix 2.** List of Included Articles

This supplemental material has been provided by the authors to give readers additional information about their work.

© 2023 Kamran R et al. *JAMA Network Open.*

**eAppendix 1.** Search Strategy

*The rows in each table below were combined with the AND Function*

| PUBMED |
|---|
| (((((((("Patient Outcome Assessment"[Mesh] OR HR-PRO[tiab] OR HRPRO[tiab] OR HRQL[tiab] OR HRQoL[tiab] OR QL[tiab] OR QoL[tiab] OR quality of life[tw] OR life quality[tw] OR health index*[tiab] OR health indices[tiab] OR health profile*[tiab] OR health status[tw] OR ((patient[tiab] OR self[tiab] OR child[tiab] OR parent[tiab] OR carer[tiab] OR proxy[tiab]) AND ((report[tiab] OR reported[tiab] OR reporting[tiab]) OR (rated[tiab] OR rating[tiab] OR ratings[tiab]) OR based[tiab] OR (assessed[tiab] OR assessment[tiab] OR assessments[tiab]))) OR ((disability[tiab] OR function[tiab] OR functional[tiab] OR functions[tiab] OR subjective[tiab] OR satisfaction[tiab] OR implementation[tiab] OR health services[tiab] OR utility[tiab] OR utilities[tiab] OR wellbeing[tiab] OR well being[tiab]) AND (index[tiab] OR indices[tiab] OR instrument[tiab] OR instruments[tiab] OR measure[tiab] OR measures[tiab] OR questionnaire[tiab] OR questionnaires[tiab] OR profile[tiab] OR profiles[tiab] OR scale[tiab] OR scales[tiab] OR score[tiab] OR scores[tiab] OR status[tiab] OR survey[tiab] OR surveys[tiab] OR PROM [tiab]))))) |
| ("Transsexualism"[Mesh] OR "Transvestism"[Mesh] OR "Transgender Persons"[Mesh] OR "Sex Reassignment Procedures"[Mesh] OR "Health Services for Transgender Persons"[Mesh] OR "Gender Identity"[Mesh] OR "Gender Dysphoria"[Mesh]) OR (transgender*[tw] OR transsexual*[tw] OR "gender identit*"[tw] OR "sex reassignment*"[tw] OR "gender dysphori*"[tw] OR "gender euphori*"[tw] OR "trans men"[tw] OR "trans man"[tw] OR "trans women"[tw] OR "trans woman"[tw] OR "cross gender*"[tw] OR crossgender*[tw] OR "trans people"[tw] OR "trans person*"[tw] OR "gender divers*"[tw] OR "gender affirm*"[tw] OR "gender incongruenc*"[tw] OR "trans people"[tw] OR "trans person"[tw] OR "gender reassignment*"[tw] OR "gender change*"[tw] OR "gender transition*"[tw] OR "trans male*"[tw] "trans masc*"[tw] OR transmasc*[tw] OR "trans female*"[tw] OR transfemin*[tw] OR "trans wom*"[tw] OR "trans man"[tw] OR "gender non-conform*"[tw] OR "gender nonconform*"[tw] OR "gender quest*"[tw] OR "two spirit*"[tw] OR "two-spirit*"[tw] OR "gender fluid*"[tw] OR genderfluid[tw] OR "non-binary"[tw] OR "nonbinary" [tw] OR agender*[tw] OR bigender*[tw] OR intergender[tw] OR "inter-gender"[tw] OR "gender varian*"[tw] OR "gender minorit*"[tw] OR "male to female"[tw] OR "female to male"[tw] OR intersex[tw] OR "inter-sex"[tw] OR multigender[tw] OR "multi-gender"[tw] OR neutrois[tw] OR novidgend*[tw] OR pangend*[tw] OR polygend*[tw] OR "third gender*"[tw] OR "gender queer"[tw] OR genderqueer[tw] OR "gender-queer"[tw]) |
| ("Sex Reassignment Procedures"[Mesh] OR "Health Services for Transgender Persons"[Mesh]) OR ("Health service*"[tw] OR "gender affirm*"[tw] OR "gender confirm*"[tw] OR "sex* reassign*"[tw] OR "sex transform*"[tw] OR "gender reassign*"[tw] OR "gender re-assign*"[tw] OR "sex confirm*"[tw] OR "sex affirm*"[tw] OR "recon* surg*"[tw] OR "breast endoprosthesis"[tw] OR "thorax surgery"[tw] OR "prophylactic mastectomy"[tw] OR "simple mastectomy"[tw] OR "partial mastectomy"[tw] OR "subcutaneous mastectomy"[tw] OR "extended radical mastectomy"[tw] OR "modified radical mastectomy"[tw] OR mastectomy[tw] OR mastectomies[tw] OR "chest contour*"[tw] OR "chest-wall contour*"[tw] OR "male genital system surgery"[tw] OR "penile recon*"[tw] OR "penile construct*"[tw] OR "vagina* reconstruction"[tw] OR "vagina* construction"[tw] OR "estrogen therap*"[tw] OR "estrogen replace*"[tw] OR "hormon* therapy"[tw] OR "urolog* surg*"[tw] OR "obstet* surg*"[tw] OR "gyne* surg*"[tw] OR feminization[tw] OR "fac* femin*"[tw] OR masculinization[tw] OR "fac* masculin*"[tw] OR "voice mod*"[tw] OR "tracheal cartilage shave" [tw] OR "otorhinolaryngolog* surg*"[tw] OR "otolaryngolog* surg*"[tw] OR "(Appearance[tw] AND physical[tw] OR facial[tw] OR face[tw] OR brow*[tw] OR cheek*[tw] OR nose[tw] OR nasal[tw] OR lip[tw] OR lips[tw] OR jaw[tw] OR jawline[tw] OR head[tw] OR forehead[tw] OR "frontal sinus"[tw] OR orbit*[tw] OR mandib*[tw] OR "mandibular angle"[tw] OR "upper lip*"[tw] OR hairline[tw] OR thyroid[tw] OR "thyroid cartilage"[tw] OR hair[tw] OR chest[tw] OR breast*[tw] OR nipple*[tw] OR hand*[tw] OR abdomen[tw] OR hips[tw] OR ribs[tw] OR rib[tw] OR ribcage[tw] OR buttocks[tw] OR thigh*[tw] OR leg[tw] OR "upper extremity"[tw] OR "lower extremity"[tw] body*[tw] OR genital*[tw] OR penis[tw] OR penile[tw] OR phallus[tw] OR "glans penis"[tw] scrotum*[tw] OR vagina[tw] OR clitori*[tw] OR vulva[tw] OR labia*[tw] OR perineum[tw] OR perineal[tw] OR body[tw] OR outward[tw] OR gender[tw] OR "gender related" [tw]) OR ("Body image"[Mesh] OR "body image"[tw] OR "body schema*"[tw] OR "body identit*"[tw] OR "body representation"[tw]) OR ("Body dissatisfaction"[Mesh] OR "body dissatisfaction"[tw] OR "appearance dissatisfaction"[tw] OR "body dismorph*"[tw]) OR ("Psychosocial Functioning"[Mesh] OR "Social participation"[Mesh] OR "Emotions"[Mesh] OR "Sexual Dysfunctions, Psychological"[Mesh] OR "Sexual health"[Mesh] OR "Mental health"[Mesh]) OR ("Sex Reassignment Surgery"[Mesh] OR (sex[tw] AND change[tw]) OR (gender[tw] AND (change[tw] OR confirm*[tw] OR affirm*[tw] OR reassignment*[tw])) OR ("Hormone Replacement Therapy"[Mesh] OR "Gonadal Steroid Hormones"[Mesh] OR "Hormones, Hormone Substitutes, and Hormone Antagonists"[Mesh] OR (steroid[tw] AND (sex[tw] OR gender[tw] OR hormone*[tw] OR gonad*[tw]) OR estrogen[tw] OR estradiol[tw] OR progesterone[tw] OR testosterone[tw] OR (hormone*[tw] AND (substitute*[tw] OR antagonist*[tw] OR inhibitor*[tw] OR modulator*[tw] OR agent*[tw] OR replace*[tw] OR therap*[tw] or management[tw])) OR ("Rehabilitation"[Mesh] OR "Activities of Daily Living"[Mesh] OR "Functional Status"[Mesh] OR "Physical functional performance"[Mesh] or "functional performance*"[tw] OR "physical performance*"[tw] OR "physical function*"[tw] OR "daily living*"[tw] OR "daily activit*"[tw] OR "Physical Therapy Modalities"[Mesh] OR physiotherap*[tw] OR "physical therap*"[tw] OR "Occupational Therapy"[Mesh] OR |

© 2023 Kamran R et al. *JAMA Network Open.*

| |
|---|
| “occupational therap*”[tw] OR “Speech Therapy”[Mesh] OR “Voice Training”[Mesh] OR “Psychotherapy”[Mesh] OR “Person-Centered Psychotherapy”[Mesh] OR “Counseling”[Mesh] OR “Mental Health Services”[Mesh] OR “Community Health Services”[Mesh] OR “Psychosocial intervention”[Mesh] OR “quality of life”[Mesh] OR “health related quality of life”[tw]) OR “Plastic Surgery”[tw] OR “cosmetic surg*”[tw] OR “esthetic surg*”[tw] OR “plastic surg*”[tw] OR “body contouring”[Mesh] OR “body lift* surg*”[tw] OR “body contour*”[tw] OR “Mammaplasty”[Mesh] OR breast recon* OR mammaplast*[tw] OR mammoplasty*[tw] OR (“breast*”[tw] AND (implant* [tw] OR endoprosthes* [tw] OR augment* [tw])) OR “tissue expan*”[tw] OR thoracic wall surg* OR chest wall surg* OR Mastectomy [Mesh] OR mammectomy*[tw] OR “radical mastectomy*”[tw] OR “chest contour*”[tw] OR “chest-wall contour*”[tw] OR “chest wall contour*”[tw] OR “chest recon*”[tw] OR “breast reduction*”[tw] OR (chest[tw] AND (masculin*[tw] OR femin*[tw])) OR “top surg*”[tw] OR (genital*[tw] AND (masculin*[tw] OR femin*[tw])) OR metoidoplast*[tw] OR (“penile” [tw] AND (construct* [tw] OR recon*[tw])) OR phalloplast*[tw] OR (penis[tw] AND (construct*[tw] OR reconst*[tw])) OR (testicular [tw] AND (construct*[tw] OR reconst*[tw] OR implant[tw] OR prosthetic[tw])) OR (testicle[tw] AND (construct*[tw] OR reconst*[tw] OR implant[tw] OR prosthetic[tw])) OR (“vagin*”[tw] AND (construct*[tw] OR recon* [tw])) OR vaginoplast*[tw] OR neophalloplast*[tw] OR “neo-phalloplast*”[tw] OR neovagin*[tw] OR neo-vagin*[tw] OR labiaplast*[tw] OR “bottom surg*”[tw] OR (fac*[tw] AND (masculin*[tw] OR femin*[tw])) scrotoplast*[tw] OR “voice surg*”[tw] OR “vocal surg*”[tw] OR thyrochondroplast*[tw] OR “tracheal cartilage”[tw] OR hysterectomy*[tw] OR orchiectom*[tw] OR ovariectom*[tw] OR salpingoophorectom*[tw] OR salpingo-oophorect*[tw] OR vaginectom*[tw] OR electrolysis[tw] OR “otolaryngology”[tw] OR castration[tw] OR gonadectomy[tw] OR genioplasty[tw] OR “hair transplant*”[tw] OR “hair procedur*”[tw] or “laser hair removal”[tw] OR “fat grafting”[tw] OR rhinoplast*[tw] |
| NOT ((“addresses”[Publication Type] OR “biography”[Publication Type] OR “case reports”[Publication Type] OR “comment”[Publication Type] OR “directory”[Publication Type] OR “editorial”[Publication Type] OR “festschrift”[Publication Type] OR “interview”[Publication Type] OR “lectures”[Publication Type] OR “legal cases”[Publication Type] OR “legislation”[Publication Type] OR “letter”[Publication Type] OR “news”[Publication Type] OR “newspaper article”[Publication Type] OR “patient education handout”[Publication Type] OR “popular works”[Publication Type] OR “congresses”[Publication Type] OR “consensus development conference”[Publication Type] OR “consensus development conference, nih”[Publication Type] OR “practice guideline”[Publication Type]) NOT (“animals”[MeSH Terms]) |

© 2023 Kamran R et al. *JAMA Network Open.*

| EMBASE |
| --- |
| patient-reported outcome.mp. or exp patient-reported outcome/ or (outcome assessment.mp. or exp outcome assessment/) or (quality of life.mp. or exp "quality of life"/) or (hr-pro or hrpro or hrql or HRQOL or ql or qol or life quality).mp. or (health index or health indices or health profile* or health status*).mp. or (appraisal* or appraised or report or reported or reporting or rated or rating* or based or assessed or assessment*).mp. or (disability or function or functional or functions or subjective or utility or utilities or wellbeing or 'well being').mp. or (index or indices or instrument or instruments or measure or measures or questionnaire* or profile or profiles or scale or scales or score or scores or status or survey or surveys).mp. OR exp health survey/ OR exp health care survey/ OR exp short survey/ OR exp health services research/ OR exp implementation science/ or implementation.mp.. |
| transgender*.tw. OR transsexual*.tw. OR ("trans m#n" or "trans wom#n" or "trans person*" or transpeople or "trans male*" or "trans female*").tw. OR ((gender adj3 (non-binary or nonbinary or bigender or agender or fluid* or trans* or non-conform* or nonconform* or genderqueer)) or divers* or incongruenc* or affirm*) or gender-nonconform*.tw. OR gender dysphori*.mp. OR two-spirit.mp. OR two spirit.mp. OR trigend*.mp. OR tri-gend*.mp. OR third gender.mp. OR gender euphoria.tw. OR (sex reassignment* or gender reassignment* or gender affirm*).tw. OR (transfeminine or transmasculine).tw. OR (AFAB or AMAB).tw. OR (gender adj (expression* or transition*)).tw. OR (transvestite* or transvestism).tw. OR exp transgender/ OR exp transsexualism/ OR exp sex reassignment/ OR exp sex transformation/ OR exp male to female transgender/ OR exp female to male transgender/ OR exp gender dysphoria/ OR ((intergender or inter gender or intersex or inter-sex or multigender or multi-gender or neutrois or novigend* or pangend* or polygend*).tw.) |
| exp physical appearance/ or exp body image/ or body image.mp. or (appearance adj3 (head or hair or hairline or frontal or facial or face or eyebrow or eye or orbit or nose or forehead or mandib* or nasal or lips or lip or cheek* or jawline or jaw or shoulders or chest or breast* or nipple* or hand* or feet or abdomen or hips or ribs or ribs or ribcage or buttocks or thigh* or leg or upper extremity or lower extremity or body* or genital* or penis or penile or phallus or glans penis or glans or scrot* or vagina or vulva or labia* or perineum or perineal or outward or gender or gender related)).mp. or health-related quality of life.mp. or exp "quality of life"/ or exp wellbeing/ or exp psychological well-being/ or exp mental health/ or exp wellbeing/ or psychosocial.mp. or exp psychosocial rehabilitation/ or exp psychosocial care/ or sexual function.mp. or exp sexual function/ or exp patient counseling/ or counseling.mp. or exp sexual counseling/ or rehabilitation.mp. or exp community based rehabilitation/ or exp rehabilitation/ or exp rehabilitation patient/ or exp speech rehabilitation/ or exp "speech and language rehabilitation"/ or exp rehabilitation care/ or exp psychosocial rehabilitation/ or physiotherapy.mp. or exp physiotherapy/ or occupational therapy.mp. or exp occupational therapy/ or voice.mp. or exp voice analysis/ or exp voice/ or voice prosthesis/ or voice training/ or exp voice change/ or hormone replacement.mp. or exp hormone substitution/ or steroid hormone/ or exp sex hormone/ or exp hormone inhibition/ or exp "silencing mediator of retinoid and thyroid hormone receptor"/ or exp hormone antagonist/ or reconstructive surgery.mp. or exp reconstructive surgery/ or exp plastic surgery/ or cosmetic surgery.mp. or exp esthetic surgery/ or (femin* adj3 (head or hair or hairline or facial or face or forehead or frontal or eyebrow or eye or orbit or nose or nasal or lips or lip or jawline or jaw or mandible or mandibular or shoulders or chest or breast* or nipple* or hand* or abdomen or hips or ribs or ribs or ribcage or buttocks or thigh* or leg or upper extremity or lower extremity or body* or genital* or outward or gender)).mp. or (masculin* adj3 (head or hair or hairline or facial or face or forehead or frontal or eyebrow or eye or orbit or nose or nasal or lips or lip or jawline or jaw or mandible or mandibular or shoulders or chest or breast* or nipple* or hand* or abdomen or hips or ribs or ribs or ribcage or buttocks or thigh* or leg or upper extremity or lower extremity or body* or genital* or outward or gender)).mp. or (viriliz* adj3 (head or hair or hairline or facial or face or forehead or frontal or eyebrow or eye or orbit or nose or nasal or lips or lip or jawline or jaw or mandible or mandibular or shoulders or chest or breast* or nipple* or hand* or abdomen or hips or ribs or ribs or ribcage or buttocks or thigh* or leg or upper extremity or lower extremity or body* or genital* or outward or gender)).mp. or (reconst* adj3 (head or hair or hairline or facial or face or forehead or frontal or eyebrow or eye or orbit or nose or nasal or lips or lip or jawline or jaw or mandible or mandibular or shoulders or chest or breast* or nipple* or hand* or abdomen or hips or ribs or ribs or ribcage or buttocks or thigh* or leg or upper extremity or lower extremity or body* or genital* or outward or gender)).mp. or (construct* adj3 (head or hair or hairline or facial or face or forehead or frontal or eyebrow or eye or orbit or nose or nasal or lips or lip or jawline or jaw or mandible or mandibular or shoulders or chest or breast* or nipple* or hand* or abdomen or hips or ribs or ribs or ribcage or buttocks or thigh* or leg or upper extremity or lower extremity or body* or genital* or penile or vaginal or outward or gender)).mp. or (breast* adj (augment* or implant* or reduction* or lift* or endoprosthes*)).mp. or (mastectom* or mammaplast* or mammoplast*).mp. or (top surg* or bottom surg* or phalloplast* or scrotoplast* or glans plast* or glansplast* or erectile device* or vaginoplast* or neophalloplast* or neo-phalloplast* or neovagin* or labiaplast* or urolog* surg* or obstet* surg* or gyne* surg* or voice surg* or vocal surg* or thyrochondroplast* or tracheal cartilage shave or hysterectomy* or orchiectom* or ovariectom* or salpingoophorectom* or vaginectom* or electrolysis or otorhinolaryngolog* surg* or otolaryngolog* surg* or castration or gonadectomy or laryngoplast* or rhinoplast* or geniolast* or hair* advanc* or hair* transplant* or laser hair removal or testicul* implant* or testicul* prosthetic*).mp. |

© 2023 Kamran R et al. *JAMA Network Open.*

| MEDLINE |
| --- |
| exp Surveys and Questionnaires/ or exp patient reported outcome measures/ or patient reported outcome measur*.mp. or patient-reported outcome.mp. or outcome assessment.mp. or HR-PRO.mp. or HRPRO.mp. or HRQL.mp. or HRQOL.mp. or QL.mp. or QOL.mp. or quality of life.mp. or function*.mp. or satisfaction.mp. or index.mp. or indices.mp. or instrument*.mp. or measure*.mp. or PROM.mp. or survey*.mp. or questionnaire*.mp. or outcome assessment.mp. or exp Health Surveys/ or exp Health Care Surveys/ or exp Outcome Assessment, Health Care/ OR exp health services administration/ OR exp implementation science/ OR patient education.mp OR implementation.mp OR exp delivery of health care/ OR exp evaluation research/ OR decision making.mp. OR framework.mp. OR diffusion of innovation.mp. |
| exp Sexual and Gender Minorities/ OR (exp Transgender Persons/ or exp Transsexualism/ or exp Gender Dysphoria/ or exp Disorders of Sex Development/ or exp Gender Identity/ or transgender*.mp. or transsexual*.mp. or transvestite*.mp. or transvestism.mp. or gender dysphoria.mp. or gender euphoria.mp. or MTF.mp. or FTM.mp. or trans m#n.mp. or trans wom#n.mp. or trans person*.mp. or transpeople.mp. or trans male*.mp. or trans female*.mp. or nonbinary.mp. or non-binary.mp. or genderqueer.mp. or gender-queer.mp. or agender.mp. or bigender.mp. or gender fluid.mp. or gender var*.mp. or two-spirit.mp. or two spirit.mp. or gender nonconform*.mp. or gender quest*.mp. or gender varian*.mp. or genderfluid.mp. or intergender.mp. or inter gender.mp. or intersex.mp. or inter sex.mp. or inter-sex.mp. or multigender.mp. or multi-gender.mp. or neutrois.mp. or novigend*.mp. or pangend*.mp. or polygend*.mp. or third gender.mp. or transfemin*.mp. or third gend*.mp. or transmasc*.mp. or trans fem*.mp. or trans masc*.mp. or transmasc*.mp. or transfemin* trigend*.mp. or AFAB.mp. or AMAB.mp. or gender expression.mp. or gender transition.mp. or gender identity.mp. or tri gend*.mp.) OR (gender adj3 (non-binary or agender or fluid* or trans* or non-conform* or nonconform*)).mp |
| exp Health Services/ or exp Health Services for Transgender Persons/ or health services for transgender persons.mp. or exp Sex Reassignment Procedures/ or exp Sex Reassignment Surgery/ or health service*.mp. or gender affirm*.mp. or gender confirm*.mp. or sex reassign*.mp. or sex transform*.mp. or gender reassign.mp. or gender re-assign*.mp. or sex confirm*.mp. or sex affirm*.mp. or exp Reconstructive Surgical Procedures/ or reconstructive surg*.mp. or exp Surgery, Plastic/ or plastic surg*.mp. or exp body contouring/ or body contour*.mp. or breast recon*.mp. or exp Mammaplasty/ or exp Breast Implants/ or exp Breast Implantation/ or mammaplasty.mp. or mammoplasty.mp. or breast implant*.mp. or breast augment*.mp. or tissue expan*.mp. or thoracic wall surg*.mp. or chest wall surg*.mp. or mastectomy.mp. or chest contour*.mp. or chest-wall contour*.mp. or chest wall contour*.mp. or reduction mammoplasty.mp. or reduction mammaplasty.mp. or mastoplasty.mp. or mastectomy.mp. or mastectomies.mp. or exp Mammaplasty/ or exp Mastectomy, Simple/ or exp Mastectomy, Segmental/ or exp Mastectomy, Modified Radical/ or exp Mastectomy/ or exp Mastectomy, Extended Radical/ or exp Mastectomy, Subcutaneous/ or exp Mastectomy, Radical/ or metoidoplast* or penile recon* or penile construct* or phalloplast* or vagina* reconstruct* or vaginoplast* or vagina* construct* or neophalloplast* or neo-phalloplast* or neovagina* or neo-vagin* or genital reassign* or genital re-assign* or exp Otolaryngology/ or otorhinolaryngolog* surg*.mp. or otolaryngolog* surg*.mp. or exp Estrogen Replacement Therapy/ or exp Hormones/ or exp Steroids/ or exp Urologic Surgical Procedures/ or estrogen replace*.mp. or testosterone therap*.mp. or urolog* surg*.mp. or obstet* surg*.mp. or gyne* surg*.mp. or fac* femin*.mp. or masculinization.mp. or top surg*.mp. or bottom surg*.mp. or fac* masculin*.mp. or exp Feminization/ or scrotoplast*.mp. or voice surg*.mp. or thyrochondroplast*.mp. or tracheal cartilage shave.mp. or exp Hysterectomy, Vaginal/ or exp Hysterectomy/ or exp orchiectomy/ or exp ovariectomy/ or exp salpingo-oophorectomy/ or vaginectomy.mp. or exp obstetric surgical procedures/ or exp Gynecologic Surgical Procedures/ or exp electrolysis/ or hysterectomy.mp. or orchiectom*.mp. or ovariectom*.mp. or salpingo-oophorect*.mp. or salpingoophorectom*.mp. or vaginectom*.mp. or voice mod*.mp. or exp electrolysis/ or electrolysis.mp. |

© 2023 Kamran R et al. *JAMA Network Open.*

| PSYCINFO |
| --- |
| exp Surveys/ OR exp Questionnaires/ OR (exp "Quality of Life"/ or exp Treatment Outcomes/ or exp Self-Report/ or exp Psychometrics/ or exp Patient Reported Outcome Measures/ or Measurement/) OR (hr-pro or hrpro or hrql or hrqol or ql or qol or life quality or satisfaction or health index or health indices or health profile* or health status).mp. OR (apprais* or report or reported or reporting or rated or rating* or based or assessed or assessment*).mp. OR (disability or function or functional or functions or subjective or utility or utilities or wellbeing or well being).mp. OR (index or indices or instrument or instruments or measure or measures or questionnaire* or profile or profiles or scale or scales or score or scores or status or survey or surveys or self-report or patient reported outcome measure* or patient-reported outcome measure or PROM*).mp. OR implementation.mp. |
| exp Health Care Delivery/ or exp Health Care Utilization/ or exp Health Care Services/ or exp Gender Reassignment/ or exp Surgery/ or exp Plastic Surgery/ OR exp Cosmetic Techniques/ or exp Mastectomy/ OR exp Drug Therapy/ or exp Steroids/ or exp Prostheses/ or exp Hysterectomy/ or exp Ovariectomy/ OR (health service* or gender affirm* or gender confirm* or sex reassign* or sex transform* or gender reassign or gender re-assign* or sex confirm* or sex affirm*) OR (reconstructive surg* or plastic surg*).mp. or exp body contouring/ or body contour*.mp. or breast recon*.mp. or mammaplasty.mp. or mammoplasty.mp. or breast implant*.mp. or breast augment*.mp. or tissue expan*.mp. or thoracic wall surg*.mp. or chest wall surg*.mp. or mastectomy.mp. or chest contour*.mp. or chest-wall contour*.mp. or chest wall contour*.mp. or reduction mammoplasty.mp. or reduction mammaplasty.mp. or mastoplasty.mp. or mastectomy.mp. or mastectomies.mp. or metoidoplast*.mp. or penile recon*.mp. or penile construct*.mp. or phalloplast*.mp. or vagina* reconstruct*.mp. or vaginoplast*.mp. or vagina* construct*.mp. or neophalloplast*.mp. or neo-phalloplast*.mp. or neovagina*.mp. or neo-vagin*.mp. or genital reassign*.mp. or genital re-assign*.mp. OR (otorhinolaryngolog* surg* or otolaryngolog* surg* or estrogen replace* or testosterone therap* or urolog* surg* or obstet* surg* or gyne* surg* or fac* femin* or masculinization or top surg* or bottom surg* or fac* masculin* or scrotoplast* or voice surg* or thyrochondroplast* or tracheal cartilage shave or vaginectomy or hysterectomy or orchiectom* or ovariectom* or salpingo-oophorect* or salpingoophorectom* or vaginectom* or voice mod* or electrolysis).mp. |
| exp Transgender/ or exp Gender Identity/ or exp Transsexualism/ or exp Gender Dysphoria/ or "Transgender (Attitudes Toward)"/ or exp Gender Reassignment/ or (trans sexual or transexual or trans m#n or trans wom#n or trans person*).mp. OR (transpeople or trans male* or trans female or sex change* or Gender Nonconform* or gender non-conform* or Gender identity or transsexualism or gender identity or non-binary or agender or bigender or trigender or tri gender or nonbinary or genderfluid* or genderqueer or gender queer or gender varian* or gender ambigui* or cross gender* or two-spirit or two spirit or gender quest* or intergender or inter gender or inter-gender or intersex or inter-sex or inter sex or multi gender or multi-gender or neutrois or novigend* or polygend* or third gender or trans masc* or transmasc* or transfemin* or trans fem*).mp. OR ((Gender adj3 (non-binary or agender or fluid* or non-conform* or nonconform*)).mp. OR exp Gender Nonconforming/ or gender euphor*.mp.) |

© 2023 Kamran R et al. *JAMA Network Open.*

| CINAHL |
| --- |
| (MH "Patient-Reported Outcomes") OR (MH "Outcome Assessment") OR (MH "Quality of Life+") OR (MH questionnaire or survey or scale or instrument or measurement or measure) OR ("TI(“hr-pro” OR “hrpro” OR “hrql” OR “hrqol” OR “ql” OR “qol” OR “quality of life” OR “life quality” OR “health index*” OR “health indices” OR “health profile*” OR “health status”) OR AB(“hr-pro” OR “hrpro” OR “hrql” OR “hrqol” OR “ql” OR “qol” OR “quality of life” OR “life quality” OR “health index*” OR “health indices” OR “health profile*” OR “health status” )OR TI( ((patient OR self OR child OR parent OR carer OR proxy) N2 (appraisal* OR appraised OR report OR reported OR reporting OR rated OR rating* OR based OR assessed OR assessment*)) ) OR AB ( ((patient OR self OR child OR parent OR carer OR proxy) N2 (appraisal* OR appraised OR report OR reported OR reporting OR rated OR rating* OR based OR assessed OR assessment*)) ) OR TI ( ((disability OR function OR functional OR functions OR subjective OR utility OR utilities OR wellbeing OR “well being”) N2 (index OR indices OR instrument OR instruments OR measure OR measures OR questionnaire* OR profile OR profiles OR scale OR scales OR score OR scores OR status OR survey OR surveys)) ) OR AB ( ((disability OR function OR functional OR functions OR subjective OR utility OR utilities OR wellbeing OR “well being”) N2 (index OR indices OR instrument OR instruments OR measure OR measures OR questionnaire* OR profile OR profiles OR scale OR scales OR score OR scores OR status OR survey OR surveys OR PROM* OR implementation OR “patient-reported outcome measure*” OR “patient reported outcome measure*” )))) |
| (MH mental health treatment or mental health services or mental health care) OR (MH psychotherapy or therapy or counseling or intervention or treatment) OR (TX psychosocial interventions or strategies or best practices or treatment or therapy) OR (MH speech therapy or treatment or intervention) OR (MH voice therapy or voice treatment or voice training or voice modification) OR TX appearance N3 ( head or hair or hairline or facial or face or forehead or frontal or eyebrow or eye or orbit or nose or nasal or lips or lip or jawline or jaw or mandib* or cheek* or shoulder* or chest or breast or nipple or hands or upper extremity or abdomen or hips or or ribs or ribcage or buttocks or thigh or leg or back or lower extremity or body or feet or genital* or penis or penile or phallus or glans penis or glans or scrot* or vagin* or vulva or labia or perineum or perineal or outward or gender or gender related or gender-related) OR TX reconstr* N3 ( head or hair or hairline or facial or face or forehead or frontal or eyebrow or eye or orbit or nose or nasal or lips or lip or jawline or jaw or mandib* or cheek* or shoulder* or chest or breast or nipple or hands or upper extremity or abdomen or hips or or ribs or ribcage or buttocks or thigh or leg or back or lower extremity or body or feet or genital* or penis or penile or phallus or glans penis or glans or scrot* or vagin* or vulva or labia or perineum or perineal or outward or gender or gender related or gender-related) OR TX construct* ( head or hair or hairline or facial or face or forehead or frontal or eyebrow or eye or orbit or nose or nasal or lips or lip or jawline or jaw or mandib* or cheek* or shoulder* or chest or breast or nipple or hands or upper extremity or abdomen or hips or or ribs or ribcage or buttocks or thigh or leg or back or lower extremity or body or feet or genital* or penis or penile or phallus or glans penis or glans or scrot* or vagin* or vulva or labia or perineum or perineal or outward or gender or gender related or gender-related) OR TX hormone blockers OR TX ( hormone replacement therapy or hrt or hormone therapy ) OR TX puberty blockers OR TX puberty hormones OR TX estrogen replacement therapy OR TX ( testosterone replacement therapy or trt ) OR TX estrogen replacement therapy OR TX ( sex hormones or estrogen or progesterone or estradiol ) OR TX hormone substitution OR TX steroid hormones OR TX hormone antagonist OR TX antiandrogen drugs OR TX spironolactone OR TX plastic surgery or cosmetic surgery or cosmetics procedures OR esthetic surgery or body contouring or mammaplasty or breast reconstruction or mammoplasty or breast implant or breast prosthesis or breast augmentation or radical mastectomy or chest contouring or chest contour or breast reduction or chest wall contour or chest reconstruction or chest masculinization or chest feminization or top surgery or bottom surgery or genital masculinization or gential feminization or genital gender affirming surgery or genital gender affirming procedures or metoidioplasty or phalloplasty or male genital reconstruction or penile reconstruction or penis reconstruction or testicular reconstruction or testicular construction or testicle construction or erectile device or erectile implant or penile prosthesis or penile implant or scrotoplasty or glansplasty or neophalloplasty or vaginal reconstruction or vaginal construction or vaginectomy or labiaplasty or clitoral reduction or clitoris or neovaginoplasty or urology or urological surgery or obstetric surgery or gynecologic surgery or facial masculinization or facial feminization or voice surgery or vocal surgery or vocal chords surgery or thyrochondroplasty or tracheal cartilage shave or hysterectomy or orchiectomy or ovariectomy or saphingoopherectomy or otorhinolaryngology or castration or gonadectomy or gemiolasty or hair transplant or hair advancement or laser hair removal or fat grafting or rhinoplasty |
| (MH "Transgender Persons+") OR (MH "Transsexuals") OR (MH "Transsexualism") OR (MH "Sex Reassignment Procedures+") OR (MH “Gender Identity”) OR (MH “Gender Dysphoria”) OR TX (trans sexual or transexual or sex change) or OR TX transgender* OR TX transsexual* OR TX "trans m#n" or "trans wom#n" or "trans person*" or transpeople or "trans male*" or "trans female*" OR TX gender* N3 (non-binary or fluid* or trans* or non-conform* |

© 2023 Kamran R et al. *JAMA Network Open.*

or nonconform* or queer or gender varian* or gender ambigui* or cross gender* or two-spirit or two spirit or gender quest* or intergender or inter gender or inter-gender or intersex or inter-sex or inter sex or multi gender or multi-gender or neutrois or novigend* or polygend* or third gender or trans masc* or transmasc* or gender identity or transsexualism or non-binary or agender or bigender or trigender or tri gender or genderfluid* or gender queer or transfemin* or trans fem* or diverse* or incongru* or affirm*) OR TX gender dysphori* OR TX sex reassignment* or gender reassignment* or gender affirm* OR TX transfeminine or transmasculine OR TX AFAB or AMAB OR TX gender N2 (expression* or transition*) OR TX gender minorit* or gender diver*

© 2023 Kamran R et al. *JAMA Network Open.*

| WEB OF SCIENCE |
| --- |
| TS=("questionnaire*" or "survey*" or "scale" or "instrument*" or "measurement" or "measure*" or "quality of life" or "life quality" or "health index*" or "health indices" or "health profile*" or "health status" or "appraisal*" or "appraised" or "report*" or "rated" or "rating*" or "based" or "assessed" or "assessment*" or "function" or "functional" or "functions" or "satisfaction" or "implantation" or "health services" or "subjective" or "utility" or "utilities" or "wellbeing" or "well being" or "index" or "indices" or "instrument" or "instruments" or "measure*" or "questionnaire*" or "profile" or "profiles" or "scale*" or "score*" or "status" or "survey*" or "PROM*" or "patient-reported outcome measure*" or "patient reported outcome measure*") |
| TS=("health service*" or "gender affirmation procedures" or "gender affirm*" or "gender confirm*" or "sex reassign*" or "sex transform*" or "gender reassign*" or "gender re-assign" or "sex confirm*" or "sex affirm*" or "gender affirmation surgery" or "gender affirming" or "surgery" or "plastic surg*" or "reconstructive surg*" or "craniofacial recon*" or "breast recon*" or "mammaplast*" or "mammoplast*" or "breast implant" or "breast augment*" or "tissue expan*" or "thoracic wall surg*" or "mastectom*" or "reduction mammoplast*" or "reduction mammaplast*" or "mastoplasty" or "metoidoplast*" or "penile recon*" or "penile construct*" or "phalloplast*" or "vagina* reconstruct*" or "vagina* construct*" or "neophalloplast*" or "neo-phalloplast*" or "neovagina" or "neo-vagina" or "genital reassign" or "genital re-assign""otorhinolaryngologic surg*" or "otolaryngologic surg*" or "head and neck surgery" or "body contour*" or "prosthesis" or "implant*" or "breast implant*" or "penile prosthesis" or "mastectom*" or "gyne* surg*" or "obstet* surg*" or "urolog* surg*" or "hormone* therap*" or "estrogen" or "testosterone" or "steroids" or "chest wall surg*" or "chest-wall surg*" or "voice therapy" or "feminization" or "hysterectomy" or "oophorectomy" or "orchiectomy" or "electrolysis" or "face feminin*" or "masculinization" or "top surg*" or "bottom surg*" or "fac* masculin*" or "scrotoplast*" or "voice surg*" or "thyrochondroplast*" or "tracheal cartilage shave" or "vaginectomy" or "hysterectomy" or "orchiectomy*" or "ovariectomy*" or "salpingo-oophorect*" or "salpingoophorectom*" or "vaginectom*" or "voice mod" or "electrolysis") |
| TS=("Transgender*" or "transsexual*" or "sex reassign*" or "gender identity" or "gender dysphoria" or "trans sexual" or "trans m#n" or "trans wom#n" or "trans person" or "transpeople" or "trans male" or "trans female" or "sex change*" or "gender nonconform*" or "gender non-conform*" or "gener nonbinary" or "non-binary" or "nonbinary" or "agender" or "bigender" or "trigender" or "tri gender" or "gender fluid" or "genderfluid" or "genderqueer" or "gender queer" or "intergender" or "inter gender" or "inter-gender" or "intersex" or "inter-sex" or "inter sex" or "multi gender" or "multi-gender" or "neutrois" or "novigend*" or "polygend*" or "third gender*" or "trans masc*" or "transmasc*" or "transfemin*" or "trans fem*" or "AFAB" or "AMAB" or "transvestit*" or "gender minorit*" or "gender diver*") |

© 2023 Kamran R et al. *JAMA Network Open.*

| Grey Literature Search Strategy | |
|---|---|
| **Database, Search Engine, or Website URL** | **Search Strategy** |
| Opengrey.eu | (Patient-reported outcome measure OR Questionnaire) AND (Transgender OR Nonbinary) AND (Gender-affirming care) AND (Implement*) |
| New York Academy of Medicine's Grey Literature Report (http://www.greylit.org/home) | (Patient-reported outcome measure OR Questionnaire) AND (Transgender OR Nonbinary) AND (Gender-affirming care) AND (Implement*) |
| National Institute for Health and Care Excellence Guidance (https://www.nice.org.uk/guidance) | (Patient-reported outcome measure OR Questionnaire) AND (Transgender OR Nonbinary) AND (Gender-affirming care) AND (Implement*) |
| Google.com (first 10 pages) | (Patient-reported outcome measure OR Questionnaire) AND (Transgender OR Nonbinary) AND (Gender-affirming care) AND (Implement*) |
| Google.co.uk (first 10 pages) | (Patient-reported outcome measure OR Questionnaire) AND (Transgender OR Nonbinary) AND (Gender-affirming care) AND (Implement*) |
| Gender Identity Research and Education Society (https://www.gires.org.uk/) | (Patient-reported outcome measure OR Questionnaire) AND (Transgender OR Nonbinary) AND (Gender-affirming care) AND (Implement*) |
| Gendered Intelligence (https://genderedintelligence.co.uk/) | (Patient-reported outcome measure OR Questionnaire) AND (Transgender OR Nonbinary) AND (Gender-affirming care) AND (Implement*) |
| Stonewall UK (https://www.stonewall.org.uk/) | (Patient-reported outcome measure OR Questionnaire) AND (Transgender OR Nonbinary) AND (Gender-affirming care) AND (Implement*) |

© 2023 Kamran R et al. *JAMA Network Open.*

**eTable 1.** Overview of Countries for Articles Included in Systematic Review

| **Country/Countries** | **Number of studies** |
|---|---|
| Australia | 10 |
| Austria | 1 |
| Belgium | 4 |
| Belgium, France | 1 |
| Brazil | 9 |
| Brazil, Spain, UK, Italy, Germany, the Netherlands, USA, France, Portugal, Australia, Belgium, Israel, Denmark, Finland, Ireland, Norway, Sweden | 1 |
| Canada | 4 |
| Canada, the Netherlands | 2 |
| Chile | 2 |
| Colombia | 1 |
| Croatia | 1 |
| Czech Republic | 1 |
| Denmark | 1 |
| France | 11 |
| Germany | 29 |
| Germany, Switzerland | 1 |
| Iran | 6 |
| Iran, the Netherlands | 1 |
| Israel | 2 |
| Italy | 18 |
| Korea | 1 |
| New Zealand | 1 |
| Poland | 1 |
| Russia | 1 |
| Serbia | 2 |
| Spain | 9 |
| Sweden | 9 |
| Switzerland | 6 |
| Switzerland, Spain | 1 |
| Taiwan | 3 |
| Thailand | 3 |
| The Netherlands | 28 |
| The Netherlands, Belgium, Germany, Norway | 6 |
| The Netherlands, Belgium, Italy, Norway | 2 |
| The Netherlands, Belgium, UK, Switzerland | 1 |
| The Netherlands, Italy, Belgium | 1 |

© 2023 Kamran R et al. *JAMA Network Open.*

| | |
|---|---|
| The Netherlands, USA | 1 |
| The Netherlands, USA, Serbia, Canada, Finland, France, Belgium | 1 |
| Turkey | 11 |
| UK | 12 |
| USA | 72 |
| USA, Canada | 4 |
| USA, Spain | 1 |
| USA, Europe, Canada, Mexico, Australia, Asia, South America | 1 |
| USA, UK, Australia, Belgium, Canada, Germany, Finland, The Netherlands, Spain | 1 |
| Yugoslavia | 1 |

© 2023 Kamran R et al. *JAMA Network Open.*

**eTable 2.** Oxford Centre for Evidence-Based Medicine Levels of Evidence[1] for Included Studies

| Level of evidence | Number of studies |
|---|---|
| 1b: RCT | 1 |
| 2b: Cohort Study | 87 |
| 2c: Outcomes Research | 190 |
| 3b: Case-Control Study | 1 |
| 4: Case-series (and poor quality cohort and case-control studies) | 7 |

1. Jeremy Howick, Iain Chalmers, Paul Glasziou, Trish Greenhalgh, Carl Heneghan, Alessandro Liberati, Ivan Moschetti, Bob Phillips, and Hazel Thornton. "Explanation of the 2011 Oxford Centre for Evidence-Based Medicine (OCEBM) Levels of Evidence (Background Document)". Oxford Centre for Evidence-Based Medicine. https://www.cebm.ox.ac.uk/resources/levels-of-evidence/explanation-of-the-2011-ocebm-levels-of-evidence/

**eTable 3.** Data Collection Platform for Patient-Reported Outcome Measure (PROM) Administration in Gender-Affirming Care

| Data collection platform | Number of studies, N/286 (%) |
|---|---|
| NR | 258 (90) |
| "Secure website" | 1 (1) |
| Electronic medical record (EMR) | 4 (1) |
| Patient chart | 1 (1) |
| Qualtrics | 7 (2) |
| Redcap | 7 (2) |
| SurveyMonkey | 6 (2) |
| University online survey management system | 1 (1) |
| Castor electronic data capture | 1 (1) |

© 2023 Kamran R et al. *JAMA Network Open.*

**eTable 4.** Data Security for Patient-Reported Outcome Measures (PROMs) in Gender-Affirming Care

| **Data security** | **Number of studies, N/286 (%)** |
|---|---|
| "Online secure platform" | 2 (1) |
| "Secure website" | 2 (1) |
| "Secured format" for "web-surveys" | 1 (1) |
| NR | 278 (97) |
| Locked box | 1 (1) |
| Restricted access/encrypted drive on the hospital server for later analysis | 1 (1) |
| The survey was provided online through a secured server using Encrypting File System technology (http://www.unipark.com/en/survey-software/). | 1 (1) |

**eTable 5.** Patient-Reported Outcome Measure (PROM) Score Reporting in Gender-Affirming Care

| **PROM score reporting method** | **Frequency, N/286 (%)** |
|---|---|
| Mean and SD in a table or written out | 162 (57) |
| Proportion of individuals at cut-off value for PROM | 55 (19) |
| Bar chart of scores | 21 (7) |
| Narrative description of scores | 17 (6) |
| Line graph of scores at different time points | 14 (5) |
| Raw scores for each individual | 5 (1) |
| Box plot of scores | 4 (1) |
| Regression with demographic factors | 3 (1) |

© 2023 Kamran R et al. *JAMA Network Open.*

| | |
|---|---|
| Median and IQR | 2 (1) |
| Pie chart | 2 (1) |
| NR – scores not reported | 1 (1) |

**eTable 6.** Patient-Reported Outcome Measure (PROM) Result Application in Gender-Affirming Care

| **How were PROM results used?** | **Frequency, N/286 (%)** |
|---|---|
| Demonstrate patient satisfaction with treatment* | 71 (25) |
| Demonstrate treatment outcomes | 187 (65) |
| Develop/validate a PROM | 28 (10) |

**Demonstration of patient satisfaction was identified through analysing conclusions made from study authors based on PROM results. For example, if study authors stated that the PROM results demonstrate patients are satisfied or dissatisfied with treatment.*

**eTable 7.** Critical Appraisal Skills Programme (CASP) Checklist Results for Included Articles

| **CASP Checklist Item** | **Rating** | | |
|---|---|---|---|
| | **Yes** | **No** | **Can't Tell** |
| *Cohort Studies Checklist (n=285)* | | | |
| Did the study address a clearly focused issue? | 285 | 0 | 0 |
| Was the cohort recruited in an acceptable way? | 275 | 2 | 8 |
| Was the exposure accurately measured to minimise bias? | 10 | 103 | 172 |
| Was the outcome accurately measured to minimise bias? | 5 | 102 | 178 |
| Have the authors identified all important | 4 | 127 | 154 |

© 2023 Kamran R et al. *JAMA Network Open.*

| | | | |
|---|---|---|---|
| confounding factors? | | | |
| Have they taken account of the confounding factors in the design and/or analysis? | 2 | 151 | 132 |
| Was the follow up of subjects complete enough? | 121 | 158 | 6 |
| Was the follow up of subjects long enough? | 88 | 193 | 4 |
| Do you believe the results? | 283 | 0 | 2 |
| Can the results be applied to the local population? | 282 | 0 | 3 |
| Do the results of this study fit with other available evidence? | 284 | 0 | 1 |
| *RCT Checklist (n=1)* | | | |
| Did the study address a clearly focused research question? | 1 | 0 | 0 |
| Was the assignment of participants to interventions randomised? | 1 | 0 | 0 |
| Were all participants who entered the study accounted for at its conclusion? | 1 | 0 | 0 |
| Were the participants ‘blind’ to intervention they were given? | 0 | 0 | 1 |
| Were the investigators ‘blind’ to the intervention they were giving to participants? | 0 | 0 | 1 |
| Were the people assessing/analysing outcome/s ‘blinded’? | 0 | 0 | 1 |

© 2023 Kamran R et al. *JAMA Network Open.*

| | | | |
|---|---|---|---|
| Were the study groups similar at the start of the randomised controlled trial? | 0 | 0 | 1 |
| Apart from the experimental intervention, did each study group receive the same level of care (that is, were they treated equally)? | 1 | 0 | 0 |
| Were the effects of intervention reported comprehensively? | 1 | 0 | 0 |
| Was the precision of the estimate of the intervention or treatment effect reported? | 1 | 0 | 0 |
| Do the benefits of the experimental intervention outweigh the harms and costs? | 1 | 0 | 0 |
| Can the results be applied to your local population/in your context? | 1 | 0 | 0 |
| Would the experimental intervention provide greater value to the people in your care than any of the existing interventions? | 0 | 0 | 1 |

© 2023 Kamran R et al. *JAMA Network Open.*

**eAppendix 2.** List of Included Articles


1. Lindgren TW, Pauly IB. A body image scale for evaluating transsexuals. *Arch Sex Behav*. 1975;4(6):639-656.

2. Becker I, Auer M, Barkmann C, et al. A Cross-Sectional Multicenter Study of Multidimensional Body Image in Adolescents and Adults with Gender Dysphoria Before and After Transition-Related Medical Interventions. doi:10.1007/s10508-018-1278-4

3. van de Grift TC, Pigot GLS, Boudhan S, et al. A Longitudinal Study of Motivations Before and Psychosexual Outcomes After Genital Gender-Confirming Surgery in Transmen. doi:10.1016/j.jsxm.2017.10.064

4. Schaff J, Papadopulos NA. A New Protocol for Complete Phalloplasty with Free Sensate and Prelaminated Osteofasciocutaneous Flaps: Experience in 37 Patients. doi:10.1002/micr.20647

5. Costantino A, Cerpolini S, Alvisi S, Morselli PG, Venturoli S, Meriggiola MC. A Prospective Study on Sexual Function and Mood in Female-to-Male Transsexuals During Testosterone Administration and After Sex Reassignment Surgery. doi:10.1080/0092623x.2012.736920

6. Beatrice J. A psychological comparison of heterosexuals, transvestites, preoperative transsexuals, and postoperative transsexuals. *J Nerv Ment Dis*. 1985;173(6):358-365.

7. van der Sluis WB, Pigot GLS, Al-Tamimi M, et al. A Retrospective Cohort Study on Surgical Outcomes of Penile Prosthesis Implantation Surgery in Transgender Men After Phalloplasty. doi:10.1016/j.urology.2019.06.010

8. Dahl KL, Mahler LA. Acoustic Features of Transfeminine Voices and Perceptions of Voice Femininity. doi:10.1016/j.jvoice.2019.05.012

9. Brown SK, Chang J, Hu S, et al. Addition of Wendler Glottoplasty to Voice Therapy Improves Trans Female Voice Outcomes. doi:10.1002/lary.29050

10. Smith YL, van Goozen SH, Cohen-Kettenis PT. Adolescents with gender identity disorder who were accepted or rejected for sex reassignment surgery: a prospective follow-up study. *J Am Acad Child Adolesc Psychiatry*. 2001;40(4):472-481.

11. Buncamper ME, Honselaar JS, Bouman MB, Özer M, Kreukels BP, Mullender MG. Aesthetic and Functional Outcomes of Neovaginoplasty Using Penile Skin in Male-to-Female Transsexuals. *J Sex Med 2015 Jul;12(7):1626-34 doi: 10 1111/jsm 12914 Epub 2015 Jun 12*. (7):1626-1634.

12. Turan S, Poyraz CA, Saglam NGU, et al. Alterations in Body Uneasiness, Eating Attitudes, and Psychopathology Before and After Cross-Sex Hormonal Treatment in Patients with Female-to-Male Gender Dysphoria. doi:10.1007/s10508-018-1189-4

13. Hancock AB. An ICF Perspective on Voice-related Quality of Life of American


© 2023 Kamran R et al. *JAMA Network Open.*

Transgender Women. doi:10.1016/j.jvoice.2016.03.013

14. Yilmaz T, Kuscu O, Sozen T, Suslu AE. Anterior Glottic Web Formation for Voice Feminization: Experience of 27 Patients. doi:10.1016/j.jvoice.2017.03.006
15. Poudrier G, Nolan IT, Cook TE, et al. Assessing Quality of Life and Patient-Reported Satisfaction with Masculinizing Top Surgery: A Mixed-Methods Descriptive Survey Study. doi:10.1097/prs.0000000000005113
16. Baldino JN, Lodge EK, Lahlou RM. Assessment of a Student-Run Free Gender-Affirming Care Clinic's Transition to Telehealth. doi:10.1089/trgh.2020.0135
17. Manrique OJ, Adabi K, Huang TC, et al. Assessment of Pelvic Floor Anatomy for Male-to-Female Vaginoplasty and the Role of Physical Therapy on Functional and Patient-Reported Outcomes. *Ann Plast Surg 2019 Jun;82(6):661-666 doi: 10 1097/SAP 0000000000001680*. (6):661-666.
18. Owen-Smith AA, Gerth J, Sineath RC, et al. Association Between Gender Confirmation Treatments and Perceived Gender Congruence, Body Image Satisfaction, and Mental Health in a Cohort of Transgender Individuals. doi:10.1016/j.jsxm.2018.01.017
19. Almazan AN, Keuroghlian AS. Association Between Gender-Affirming Surgeries and Mental Health Outcomes. doi:10.1001/jamasurg.2021.0952
20. Dacakis G, Oates J, Douglas J. Associations between the Transsexual Voice Questionnaire (TVQ(MtF) ) and self-report of voice femininity and acoustic voice measures. *Int J Lang Commun Disord 2017 Nov;52(6):831-838 doi: 10 1111/1460-6984 12319 Epub 2017 Apr 19*. (6):831-838.
21. Hardy TLD, Rieger JM, Wells K, Boliek CA. Associations Between Voice and Gestural Characteristics of Transgender Women and Self-Rated Femininity, Satisfaction, and Quality of Life. doi:10.1044/2020_ajslp-20-00118
22. Kuper LE, Mathews S, Lau M. Baseline Mental Health and Psychosocial Functioning of Transgender Adolescents Seeking Gender-Affirming Hormone Therapy. doi:10.1097/dbp.0000000000000697
23. Cai X, Hughto JMW, Reisner SL, Pachankis JE, Levy BR. Benefit of Gender-Affirming Medical Treatment for Transgender Elders: Later-Life Alignment of Mind and Body. doi:10.1089/lgbt.2017.0262
24. Kuper LE, Stewart S, Preston S, Lau M, Lopez X. Body Dissatisfaction and Mental Health Outcomes of Youth on Gender-Affirming Hormone Therapy. doi:10.1542/peds.2019-3006
25. Garz M, Schroder J, Nieder T, et al. Body Image and Sexual Desire in the Context of Gender Affirming Therapy: Results of a Cross-sectional Multi-centered Transgender Study. doi:10.1080/0092623x.2021.1888831
26. Kraemer B, Delsignore A, Schnyder U, Hepp U. Body image and transsexualism. doi:10.1159/000111554
27. van de Grift TC, Kreukels BPC, Elfering L, et al. Body Image in Transmen:

© 2023 Kamran R et al. *JAMA Network Open.*

Multidimensional Measurement and the Effects of Mastectomy. doi:10.1016/j.jsxm.2016.09.003

28. Köhler A, Becker I, Richter-Appelt H, et al. Behandlungserfahrungen und soziale Unterstützung bei Personen mit Geschlechtsinkongruenz/Geschlechtsdysphorie: Eine ENIGI 5-Jahres-Follow-Up-Studie in drei europäischen Ländern. *Psychother Psychosom Med Psychol*. 2019;69(8):339-347. doi:10.1055/a-0806-6892

29. Becker I, Nieder TO, Cerwenka S, et al. Body Image in Young Gender Dysphoric Adults: A European Multi-Center Study. *Archives of sexual behavior*. 2016;45(3):559-574. doi:10.1007/s10508-015-0527-z

30. van de Grift TC, Cohen-Kettenis PT, Steensma TD, et al. Body Satisfaction and Physical Appearance in Gender Dysphoria. *Arch Sex Behav*. 2016;45(3):575-585. doi:10.1007/s10508-015-0614-1

31. Cantini JE, Garcia-Botero A, Rubiano V, Guerrero DN, Benitez JM, Gomez-Ortega V. Breast Augmentation in Gender Dysphoria: A Surgical Option to Reduce Reintervention. doi:10.1097/prs.0000000000008172

32. Coon D, Lee E, Fischer B, Darrach H, Landford WN. Breast Augmentation in the Transfemale Patient: Comprehensive Principles for Planning and Obtaining Ideal Results. doi:10.1097/prs.0000000000006819

33. Deuster D, Di Vincenzo K, Szukaj M, Am Zehnhoff-Dinnesen A, Dobel C. Change of speech fundamental frequency explains the satisfaction with voice in response to testosterone therapy in female-to-male gender dysphoric individuals. doi:10.1007/s00405-016-4043-0

34. Cysarz D, Piwowarczyk A, Czernikiewicz W, Dulko S, Kokoszka A. Changes in body image satisfaction, sense of coherence and life satisfaction during the therapy of women with transsexualism. A preliminary report.

35. Morselli PG, Summo V, Pinto V, Fabbri E, Meriggiola MC. Chest Wall Masculinization in Female-to-Male Transsexuals Our Treatment Algorithm and Life Satisfaction Questionnaire. doi:10.1097/sap.0000000000002119

36. Aires MM, de Vasconcelos D, de Moraes BT. Chondrolaryngoplasty in transgender women: Prospective analysis of voice and aesthetic satisfaction. doi:10.1080/26895269.2020.1848690

37. Gomez-Gil E, Gomez A, Canizares S, et al. Clinical Utility of the Bem Sex Role Inventory (BSRI) in the Spanish Transsexual and Nontranssexual Population. doi:10.1080/00223891.2011.650302

38. di Summa PG, Watfa W, Krahenbuhl S, Schaffer C, Raffoul W, Bauquis O. Colic-Based Transplant in Sexual Reassignment Surgery: Functional Outcomes and Complications in 43 Consecutive Patients. doi:10.1016/j.jsxm.2019.09.007

39. Towers S, Prizgintas D, Crossen K. Community-based model for adolescent transgender health care. doi:10.1111/jpc.15570

© 2023 Kamran R et al. *JAMA Network Open.*

40. Rieger UM, Majenka P, Wirthmann A, Sohn M, Bozkurt A, Djedovic G. Comparative Study of the Free Microvascular Groin Flap: Optimizing the Donor Site After Free Radial Forearm Flap Phalloplasty. doi:10.1016/j.urology.2016.04.007
41. Mora E, Cobeta I, Becerra A, Lucio MJ. Comparison of Cricothyroid Approximation and Glottoplasty for Surgical Voice Feminization in Male-to-Female Transsexuals. doi:10.1002/lary.27172
42. Djordjevic ML, Bizic MR. Comparison of Two Different Methods for Urethral Lengthening in Female to Male (Metoidioplasty) Surgery. doi:10.1111/jsm.12108
43. Schoffer AK, Bittner AK, Hess J, Kimmig R, Hoffmann O. Complications and satisfaction in transwomen receiving breast augmentation: short- and long-term outcomes. *Archives of Gynecology and Obstetrics*. 2022;305(6):1517-1524. doi:10.1007/s00404-022-06603-3
44. Lowenberg H, Lax H, Neto RR, Krege S. Complications, subjective Satisfaction and sexual Experience by gender reassignment Surgery in Male-to-Female Transsexual. doi:10.1055/s-0030-1262718
45. Isung J, Mollermark C, Farnebo F, Lundgren K. Craniofacial Reconstructive Surgery Improves Appearance Congruence in Male-to-Female Transsexual Patients. doi:10.1007/s10508-017-1012-7
46. Matai V, Cheesman AD, Clarke PM. Cricothyroid approximation and thyroid chondroplasty: A patient survey. doi:10.1016/s0194-5998(03)00462-5
47. Yang CY, Palmer AD, Murray KD, Meltzer TR, Cohen JI. Cricothyroid approximation to elevate vocal pitch in male-to-female transsexuals: Results of surgery. doi:10.1177/000348940211100602
48. Fisher AD, Castellini G, Ristori J, et al. Cross-Sex Hormone Treatment and Psychobiological Changes in Transsexual Persons: Two-Year Follow-Up Data. doi:10.1210/jc.2016-1276
49. Giovanardi G, Mirabella M, Di Giuseppe M, Lombardo F, Speranza AM, Lingiardi V. Defensive Functioning of Individuals Diagnosed With Gender Dysphoria at the Beginning of Their Hormonal Treatment. doi:10.3389/fpsyg.2021.665547
50. Gorjian Z, Zarenezhad M, Mahboubi M, Gholamzadeh S, Mahmoudi N. Depression in patients suffering from gender dysphoria: The hospitalized patients of Legal Medicine Center in Southwest of Iran. doi:10.5742/mewfm.2017.93018
51. Gomez-Gil E, Zubiaurre-Elorza L, de Antonio IE, Guillamon A, Salamero M. Determinants of quality of life in Spanish transsexuals attending a gender unit before genital sex reassignment surgery. doi:10.1007/s11136-013-0497-3
52. Tagay S, Breidenstein A, Friederich HC, Rubben H, Teufel M, Hess J. Development and Validation of the Essen Transgender Quality of Life -

© 2023 Kamran R et al. *JAMA Network Open.*

Inventory in a Sample of Male-to-female Transgender Persons. doi:10.1055/s-0044-101502

53. Casado-Morente JC, Rivas EM, Reina CO, et al. Development and validation of the Spanish abbreviated version of the Transsexual Voice Questionnaire for Male-to-Female Transsexuals. doi:10.1016/j.otorri.2020.01.003

54. Kattari SK, Curley KM, Bakko M, Misiolek BA. Development and Validation of the Trans-Inclusive Provider Scale. doi:10.1016/j.amepre.2019.12.005

55. Kattari SK, O'Connor AA, Kattari L. Development and Validation of the Transgender Inclusive Behavior Scale (TIBS). doi:10.1080/00918369.2017.1314160

56. Klassen AF, Kaur M, Poulsen L, et al. Development of the BODY-Q Chest Module Evaluating Outcomes following Chest Contouring Surgery. *Plast Reconstr Surg 2018 Dec;142(6):1600-1608 doi: 10 1097/PRS 0000000000004978*. (6):1600-1608.

57. Verbruggen C, Weigert R, Corre P, Casoli V, Bondaz M. Development of the facial feminization surgery patient's satisfaction questionnaire (QESFF1): Qualitative phase. doi:10.1016/j.anplas.2017.12.003

58. McGuire FH, Carl A, Woodcock L, et al. Differences in Patient and Parent Informant Reports of Depression and Anxiety Symptoms in a Clinical Sample of Transgender and Gender Diverse Youth. doi:10.1089/lgbt.2020.0478

59. Lee A, Wisco JJ, Shehan JN, Basa K, Spiegel JH. Dysphagia After Gender Affirming Chondrolaryngoplasty (Tracheal Shave): A Survey Study. *Facial Plastic Surgery & Aesthetic Medicine*. Published online 2021. doi:10.1089/fpsam.2021.0214

60. Caprini RM, Oberoi MK, Dejam D, et al. Effect of Gender-affirming Facial Feminization Surgery on Psychosocial Outcomes. *Annals of Surgery*. Published online 2022. doi:10.1097/sla.0000000000005472

61. Ozata Yildizhan B, Yuksel S, Avayu M, Noyan H, Yildizhan E. Effects of Gender Reassignment on Quality of Life and Mental Health in People with Gender Dysphoria. doi:10.5080/u18259

62. Kelly V, Hertegard S, Eriksson J, Nygren U, Sodersten M. Effects of Gender-confirming Pitch-raising Surgery in Transgender Women a Long-term Follow-up Study of Acoustic and Patient-reported Data. doi:10.1016/j.jvoice.2018.03.005

63. van de Grift TC, Elaut E, Cerwenka SC, et al. Effects of Medical Interventions on Gender Dysphoria and Body Image: A Follow-Up Study. doi:10.1097/psy.0000000000000465

64. Davis SA, Meier SC. Effects of Testosterone Treatment and Chest Reconstruction Surgery on Mental Health and Sexuality in Female-To-Male Transgender People. doi:10.1080/19317611.2013.833152

65. Nygren U, Nordenskjold A, Arver S, Sodersten M. Effects on Voice Fundamental Frequency and Satisfaction with Voice in Trans Men during

© 2023 Kamran R et al. *JAMA Network Open.*

Testosterone Treatment-A Longitudinal Study. doi:10.1016/j.jvoice.2015.10.016

66. Quinn S, Swain N. Efficacy of intensive voice feminisation therapy in a transgender young offender. doi:10.1016/j.jcomdis.2018.02.001

67. Prunas A, Fisher AD, Bandini E, et al. Eudaimonic Well-Being in Transsexual People, Before and After Gender Confirming Surgery. doi:10.1007/s10902-016-9780-7

68. Singh J, Lou A, Green M, Keely E, Greenaway M, Liddy C. Evaluation of an electronic consultation service for transgender care. doi:10.1186/s12875-021-01401-3

69. Sir E, Tuluy Y. Evaluation of Life Improvement in Trans Men After Mastectomy: A Prospective Study Using the TRANS-Q. *Aesthetic Plastic Surgery*. 2022;46(5):2556-2561. doi:10.1007/s00266-022-02907-6

70. Diamant N, Amir O. Examining the voice of Israeli transgender women: Acoustic measures, voice femininity and voice-related quality-of-life. doi:10.1080/26895269.2020.1798838

71. Markovic L, McDermott DT, Stefanac S, et al. Experiences and Interactions with the Healthcare System in Transgender and Non-Binary Patients in Austria: An Exploratory Cross-Sectional Study. doi:10.3390/ijerph18136895

72. Gooren LJ, Sungkaew T, Giltay EJ. Exploration of functional health, mental well-being and cross-sex hormone use in a sample of Thai male-to-female transgendered persons (kathoeys). *Asian J Androl*. 2013;15(2):280-285. doi:10.1038/aja.2012.139

73. van Dijk D, Dekker M, Conemans EB, et al. Explorative Prospective Evaluation of Short-Term Subjective Effects of Hormonal Treatment in Trans People-Results from the European Network for the Investigation of Gender Incongruence. doi:10.1016/j.jsxm.2019.05.009

74. Peterson CM, Toland MD, Matthews A, Mathews S, Thompson F, Conard LAE. Exploring the Eating Disorder Examination Questionnaire in Treatment Seeking Transgender Youth. doi:10.1037/sgd0000386

75. Dacakis G, Oates JM, Douglas JM. Exploring the validity of the Transsexual Voice Questionnaire (male-to-female): Do TVQ(MtF) scores differentiate between MtF women who have had gender reassignment surgery and those who have not? doi:10.1080/15532739.2016.1222922

76. Davies SM, Johnston JR. Exploring the Validity of the Transsexual Voice Questionnaire for Male-to-Female Transsexuals.

77. Schmidt M, Ramelli E, Atlan M, Cristofari S. FACE-Q satisfaction following upper third facial gender-affirming surgery using custom bone-section guides. *International journal of oral and maxillofacial surgery*. Published online 2022. doi:10.1016/j.ijom.2022.11.007

78. Capitan L, Simon D, Kaye K, Tenorio T. Facial Feminization Surgery: The Forehead. Surgical Techniques and Analysis of Results. doi:10.1097/prs.0000000000000545

79. Silva DC, Salati LR, Villas-Boas AP, et al. Factors Associated With Ruminative Thinking in Individuals With Gender Dysphoria. doi:10.3389/fpsyt.2021.602293

80. Lawrence AA. Factors associated with satisfaction or regret following male-to-female sex reassignment surgery. doi:10.1023/a:1024086814364

81. Mancini I, Tarditi D, Gava G, et al. Feasibility, Safety, and Satisfaction of Combined Hysterectomy with Bilateral Salpingo-Oophorectomy and Chest Surgery in Transgender and Gender Non-Conforming Individuals. doi:10.3390/ijerph18137133

82. Motmans J, Meier P, Ponnet K, T'Sjoen G. Female and Male Transgender Quality of Life: Socioeconomic and Medical Differences. doi:10.1111/j.1743-6109.2011.02569.x

83. Bertrand B, Perchenet AS, Colson TR, Drai D, Casanova D. Female-to-male transgender chest reconstruction: A retrospective study of patient satisfaction. doi:10.1016/j.anplas.2017.05.005

84. Goddard JC, Vickery RM, Qureshi A, Summerton DJ, Khoosal D, Terry TR. Feminizing genitoplasty in adult transsexuals: early and long-term surgical results. doi:10.1111/j.1464-410X.2007.07017.x

85. Lobato MII, Koff WJ, Manenti C, et al. Follow-up of sex reassignment surgery in transsexuals: A Brazilian cohort. *Archives of Sexual Behavior*. 2006;35(6):711-715. doi:10.1007/s10508-006-9074-y

86. de Blok CJM, Staphorsius AS, Wiepjes CM, Smit JM, Nanayakkara PWB, den Heijer M. Frequency, Determinants, and Satisfaction of Breast Augmentation in Trans Women Receiving Hormone Treatment. doi:10.1016/j.jsxm.2019.10.021

87. Pavlidis L, Spyropoulou GA, Dionyssiou D, Demiri E. Full Facial Feminization Surgery: Patient Satisfaction Assessment Based on 180 Procedures Involving 33 Consecutive Patients. *Plastic and reconstructive surgery*. 2016;138(4):765e-766e. doi:10.1097/PRS.0000000000002580

88. Veerman H, de Rooij FPW, Al-Tamimi M, et al. Functional Outcomes and Urological Complications after Genital Gender Affirming Surgery with Urethral Lengthening in Transgender Men. doi:10.1097/ju.0000000000000795

89. Dacakis G, Oates JM, Douglas JM. Further Evidence of the Construct Validity of the Transsexual Voice Questionnaire (TVQ(MtF)) Using Principal Components Analysis. doi:10.1016/j.jvoice.2016.07.001

90. Liedl B, Kogler T, Witczak M, Himmler M, Wallmichrath J. Gender affirmation female to male-metaidoioplasty. doi:10.1007/s00120-020-01335-1

© 2023 Kamran R et al. *JAMA Network Open.*

91. Fontanari AMV, Vilanova F, Schneider MA, et al. Gender Affirmation Is Associated with Transgender and Gender Nonbinary Youth Mental Health Improvement. doi:10.1089/lgbt.2019.0046

92. Riquelme JB, Naser NF, Puertas JB, Kalil JA, Arevalo MC. Gender affirmation surgeries in transgender women: Aesthetic, sexual, and urinary results of an initial series of vaginoplasties. doi:10.1016/j.acuro.2020.08.007

93. Testa RJ, Rider GN, Haug NA, Balsam KF. Gender Confirming Medical Interventions and Eating Disorder Symptoms Among Transgender Individuals. doi:10.1037/hea0000497

94. Meyenburg B, Kroger A, Neugebauer R. Gender dysphoria in children and adolescents - treatment guidelines and follow-up study. doi:10.1024/1422-4917/a000332

95. Bandini E, Fisher AD, Castellini G, et al. Gender Identity Disorder and Eating Disorders: Similarities and Differences in Terms of Body Uneasiness. doi:10.1111/jsm.12062

96. Tebbens M, Nota NM, Liberton N, et al. Gender-Affirming Hormone Treatment Induces Facial Feminization in Transwomen and Masculinization in Transmen: Quantification by 3D Scanning and Patient-Reported Outcome Measures. doi:10.1016/j.jsxm.2019.02.011

97. Tomita KK, Testa RJ, Balsam KF. Gender-Affirming Medical Interventions and Mental Health in Transgender Adults. doi:10.1037/sgd0000316

98. El-Hadi H, Stone J, Temple-Oberle C, Harrop AR. Gender-Affirming Surgery for Transgender Individuals: Perceived Satisfaction and Barriers to Care. doi:10.1177/2292550318767437

99. Manrique OJ, Sabbagh MD, Ciudad P, et al. Gender-Confirmation Surgery Using the Pedicle Transverse Colon Flap for Vaginal Reconstruction: A Clinical Outcome and Sexual Function Evaluation Study. *Plast Reconstr Surg*. 2018;141(3):767-771. doi:10.1097/PRS.0000000000004122

100. Westerflier C, Meijerink W, Tuynman JB, van der Sluis WB, Bouman MB. Gender-Confirmation Surgery Using the Pedicle Transverse Colon Flap for Vaginal Reconstruction: A Clinical Outcome and Sexual Function Evaluation Study. doi:10.1097/prs.0000000000004753

101. Pigot GLS, Al-Tamimi M, Nieuwenhuijzen JA, et al. Genital Gender-Affirming Surgery Without Urethral Lengthening in Transgender Men-A Clinical Follow-Up Study on the Surgical and Urological Outcomes and Patient Satisfaction. *Journal of Sexual Medicine*. 2020;17(12):2478-2487. doi:10.1016/j.jsxm.2020.08.004

102. LeBreton M, Courtois F, Journel NM, et al. Genital Sensory Detection Thresholds and Patient Satisfaction With Vaginoplasty in Male-to-Female Transgender Women. *J Sex Med 2017 Feb;14(2):274-281 doi: 10 1016/j jsxm 2016 12 005*. (2):274-281.

© 2023 Kamran R et al. *JAMA Network Open.*

103. Fang RH, Kao YS, Ma S, Lin JT. Glans sculpting in phalloplasty - experiences in female-to-male transsexuals. doi:10.1054/bjps.1997.0220

104. Bradford NJ, Rider NG, Spencer KG. Hair removal and psychological well-being in transfeminine adults: associations with gender dysphoria and gender euphoria. doi:10.1080/09546634.2019.1687823

105. Fallahtafti E, Nasehi M, Rasuli R, Farhud DD, Pourebrahim T, Zareeeimahmoodabadi H. Happiness and Mental Health in Pre-Operative and Post-Operative Transsexual People.

106. O'Bryan J, Scribani M, Leon K, Tallman N, Wolf-Gould C, Gadomski A. Health-related quality of life among transgender and gender expansive youth at a rural gender wellness clinic. doi:10.1007/s11136-020-02430-8

107. Riggs DW, Coleman K, Due C. Healthcare experiences of gender diverse Australians: a mixed-methods, self-report survey. doi:10.1186/1471-2458-14-230

108. Gorin-Lazard A, Baumstarck K, Boyer L, et al. Hormonal Therapy Is Associated With Better Self-esteem, Mood, and Quality of Life in Transsexuals. doi:10.1097/nmd.0000000000000046

109. Ristori J, Cocchetti C, Castellini G, et al. Hormonal Treatment Effect on Sexual Distress in Transgender Persons: 2-Year Follow-Up Data. doi:10.1016/j.jsxm.2019.10.008

110. Tucker RP, Testa RJ, Simpson TL, Shipherd JC, Blosnich JR, Lehavot K. Hormone therapy, gender affirmation surgery, and their association with recent suicidal ideation and depression symptoms in transgender veterans. doi:10.1017/s0033291717003853

111. Gomez-Gil E, Zubiaurre-Elorza L, Esteva I, et al. Hormone-treated transsexuals report less social distress, anxiety and depression. doi:10.1016/j.psyneuen.2011.08.010

112. Mayer TK, Koehler A, Eyssel J, Nieder TO. How Gender Identity and Treatment Progress Impact Decision-Making, Psychotherapy and Aftercare Desires of Trans Persons. doi:10.3390/jcm8050749

113. Elfering L, van de Grift TC, Al-Tamimi M, et al. How Sensitive Is the Neophallus? Postphalloplasty Experienced and Objective Sensitivity in Transmasculine Persons. doi:10.1016/j.esxm.2021.100413

114. Cabezas RF, Zueiga MSS, Sandoval ED, Zurita TP, Bustamante FQ, Orellana MR. Immediate effect of semioccluded vocal tract therapy on acoustic parameters in the processes of masculinization and feminization of the voice. doi:10.5209/rlog.68132

115. Smalley JM, Lozano JM, McMahon CJ, Colburn JA. Improving Global Access to Transgender Healthcare: Outcomes of a Telehealth Quality Improvement Study for the Air Force Transgender Program. doi:10.1089/trgh.2020.0167

© 2023 Kamran R et al. *JAMA Network Open.*

116. Staples JM, Bird ER, Gregg JJ, George W. Improving the Gender-Affirmation Process for Transgender and Gender-Nonconforming Individuals: Associations Among Time Since Transition Began, Body Satisfaction, and Sexual Distress. doi:10.1080/00224499.2019.1617829

117. Russell MR, Rogers RL, Rosenthal SM, Lee JY. Increasing Access to Care for Transgender/Gender Diverse Youth Using Telehealth: A Quality Improvement Project. doi:10.1089/tmj.2021.0268

118. Nieder TO, Mayer TK, Hinz S, Fahrenkrug S, Herrmann L, Becker-Hebly I. Individual Treatment Progress Predicts Satisfaction With Transition-Related Care for Youth With Gender Dysphoria: A Prospective Clinical Cohort Study. doi:10.1016/j.jsxm.2020.12.010

119. Reisner SL, Pletta DR, Potter J, Deutsch MB. Initial Psychometric Evaluation of a Brief Sexual Functioning Screening Tool for Transmasculine Adults: Transmasculine Sexual Functioning Index. doi:10.1016/j.esxm.2020.05.006

120. Gumussoy S, Hortu I, Donmez S, Dal NA, Ergenoglu AM. Investigation of body image perception, self-esteem, and self-confidence in female-to-male transsexuals before and after sex reassignment surgery. doi:10.1111/ppc.12883

121. Gorin-Lazard A, Baumstarck K, Boyer L, et al. Is Hormonal Therapy Associated with Better Quality of Life in Transsexuals? A Cross-Sectional Study. doi:10.1111/j.1743-6109.2011.02564.x

122. Kim SK, Park JW, Lim KR, Lee KC. Is Rectosigmoid Vaginoplasty Still Useful? doi:10.5999/aps.2017.44.1.48

123. Yilmaz T, Ozer F, Aydinli FE. Laser Reduction Glottoplasty for Voice Feminization: Experience on 28 Patients. doi:10.1177/0003489421993728

124. Vilenchik V, Thomas K, Baker L, Hitchens E, Keith D. Laser therapy is a safe and effective treatment for unwanted hair in adults undergoing male to female sex reassignment. doi:10.1111/ced.14466

125. Orloff LA, Mann AP, Damrose JF, Goldman SN. Laser-assisted voice adjustment (LAVA) in transsexuals. doi:10.1097/01.mlg.0000205198.65797.59

126. Eldh J, Berg A, Gustafsson M. Long term follow up after sex reassignment surgery.

127. Weyers S, Elaut E, De Sutter P, et al. Long-term Assessment of the Physical, Mental, and Sexual Health among Transsexual Women. doi:10.1111/j.1743-6109.2008.01082.x

128. Timmermans FW, Elfering L, Smit JM, van de Grift TC, Bouman MB, Mullender MG. Long-Term Changes in Free Nipple Graft Morphology and Patient-Reported Outcomes in Gender-Affirming Mastectomies. *Aesthetic Plastic Surgery*. 2022;46(5):2174-2180. doi:10.1007/s00266-021-02666-w

© 2023 Kamran R et al. *JAMA Network Open.*

129. Aldridge Z, Patel S, Guo BL, et al. Long-term effect of gender-affirming hormone treatment on depression and anxiety symptoms in transgender people: A prospective cohort study. doi:10.1111/andr.12884

130. Lindemalm G, Korlin D, Uddenberg N. Long-term follow-up of “sex change” in 13 male-to-female transsexuals. *Arch Sex Behav*. 1986;15(3):187-210.

131. Ruppin U, Pfafflin F. Long-Term Follow-Up of Adults with Gender Identity Disorder. doi:10.1007/s10508-014-0453-5

132. van der Sluis WB, Bouman MB, de Boer NK, et al. Long-Term Follow-Up of Transgender Women After Secondary Intestinal Vaginoplasty. *J Sex Med 2016 Apr;13(4):702-10 doi: 10 1016/j jsxm 2016 01 008 Epub 2016 Feb 24*. (4):702-710.

133. Kanhai RCJ, Hage JJ, Mulder JW. Long-term outcome of augmentation mammaplasty in male-to-female transsexuals: a questionnaire survey of 107 patients. doi:10.1054/bjps.1999.3298

134. Leriche A, Timsit MO, Morel-Journel N, Bouillot A, Dembele D, Ruffion A. Long-term outcome of forearm flee-flap phalloplasty in the treatment of transsexualism. doi:10.1111/j.1464-410X.2007.07362.x

135. Fakin RM, Zimmermann S, Kaye K, Lunger L, Weinforth G, Giovanoli P. Long-Term Outcomes in Breast Augmentation in Trans-Women: A 20-Year Experience. doi:10.1093/asj/sjy143

136. Morrison SD, Satterwhite T, Grant DW, Kirby J, Laub DR, VanMaasdam J. Long-Term Outcomes of Rectosigmoid Neocolporrhaphy in Male-to-Female Gender Reassignment Surgery. doi:10.1097/prs.0000000000001459

137. Sigurjonsson H, Mollermark C, Rinder J, Farnebo F, Lundgren TK. Long-Term Sensitivity and Patient-Reported Functionality of the Neoclitoris After Gender Reassignment Surgery. doi:10.1016/j.jsxm.2016.12.003

138. Achille C, Taggart T, Eaton NR, et al. Longitudinal impact of gender-affirming endocrine intervention on the mental health and well-being of transgender youths: preliminary results. doi:10.1186/s13633-020-00078-2

139. Lang CL, Day DL, Klit A, Mejdahl MK, Holmgaard R. Low Risk of Persistent Pain, Sensory Disturbances, and Complications Following Mastectomy After Gender-Affirming Surgery. doi:10.1089/trgh.2020.0070

140. Papadopulos NA, Zavlin D, Lelle JD, et al. Male-to-Female Sex Reassignment Surgery Using the Combined Technique Leads to Increased Quality of Life in a Prospective Study. doi:10.1097/prs.0000000000003529

141. Chen W, Cylinder I, Najafian A, Dugi DD 3rd, Berli JU. Male-to-Female Sex Reassignment Surgery using the Combined Vaginoplasty Technique: Satisfaction of Transgender Patients with Aesthetic, Functional, and Sexual Outcomes. *Plast Reconstr Surg 2021 Feb 1;147(2):480-483 doi: 10 1097/PRS 0000000000007579*. (1):178-187.

© 2023 Kamran R et al. *JAMA Network Open.*

142. Zavlin D, Schaff J, Lelle JD, et al. Male-to-Female Sex Reassignment Surgery using the Combined Vaginoplasty Technique: Satisfaction of Transgender Patients with Aesthetic, Functional, and Sexual Outcomes. doi:10.1007/s00266-017-1003-z

143. Bar MA, Jarus T, Wada M, Rechtman L, Noy E. Male-to-female transitions: Implications for occupational performance, health, and life satisfaction. *Can J Occup Ther 2016 Apr;83(2):72-82 doi: 10 1177/0008417416635346*. (2):72-82.

144. Wagner S, Greco F, Hoda MR, et al. Male-to-Female Transsexualism: Technique, Results and 3-Year Follow-Up in 50 Patients. doi:10.1159/000288238

145. Meier SC, Pardo ST, Labuski C, Babcock J. Measures of Clinical Health among Female-to-Male Transgender Persons as a Function of Sexual Orientation. doi:10.1007/s10508-012-0052-2

146. Sevelius JM, Chakravarty D, Dilworth SE, Rebchook G, Neilands TB. Measuring Satisfaction and Comfort with Gender Identity and Gender Expression among Transgender Women: Development and Validation of the Psychological Gender Affirmation Scale. doi:10.3390/ijerph18063298

147. Kozee HB, Tylka TL, Bauerband LA. Measuring Transgender Individuals' Comfort With Gender Identity and Appearance: Development and Validation of the Transgender Congruence Scale. doi:10.1177/0361684312442161

148. Tordoff DM, Wanta JW, Collin A, Stepney C, Inwards-Breland DJ, Ahrens K. Mental Health Outcomes in Transgender and Nonbinary Youths Receiving Gender-Affirming Care. *Jama Network Open*. 2022;5(2):e220978-e220978. doi:10.1001/jamanetworkopen.2022.0978

149. Salvador J, Massuda R, Andreazza T, et al. Minimum 2-year follow up of sex reassignment surgery in Brazilian male-to-female transsexuals. *Psychiatry Clin Neurosci*. 2012;66(4):371-372. doi:10.1111/j.1440-1819.2012.02342.x

150. Braun H, Zhang Q, Getahun D, et al. Moderate-to-Severe Acne and Mental Health Symptoms in Transmasculine Persons Who Have Received Testosterone. doi:10.1001/jamadermatol.2020.5353

151. Ramella V, Papa G, Stocco C, Torelli L, Vasselli B, Arnez ZM. New Algorithm for Chest-wall Surgery and Quality of Life Assessment in Female-to-male Reassignment Patients. doi:10.1097/gox.0000000000003121

152. Vesely J, Hyza P, Ranno R, et al. New technique of total phalloplasty with reinnervated latissimus dorsi myocutaneous free flap in female-to-male transsexuals. *Annals of Plastic Surgery*. 2007;58(5):544-550. doi:10.1097/01.sap.0000245123.16757.15

153. Vedovo F, Di Blas L, Perin C, et al. Operated Male-to-Female Sexual Function Index: Validity of the First Questionnaire Developed to Assess Sexual Function after Male-to-Female Gender Affirming Surgery. *J Urol 2020 Jul;204(1):115-120 doi: 10 1097/JU 0000000000000791 Epub 2020 Jan 31*. (1):115-120.

© 2023 Kamran R et al. *JAMA Network Open.*

154. Bultynck C, Pas C, Defreyne J, Cosyns M, T'Sjoen G. Organizing the voice questionnaire for transgender persons. doi:10.1080/15532739.2019.1605555

155. Lief HI, Hubschman L. Orgasm in the Postoperative Transsexual. doi:10.1007/bf01542363

156. Boas SR, Ascha M, Morrison SD, et al. Outcomes and Predictors of Revision Labiaplasty and Clitoroplasty after Gender-Affirming Genital Surgery. doi:10.1097/prs.0000000000006282

157. Tirrell AR, Abu El Hawa A, Bekeny JC, Del Corral G. Outcomes in chest feminization patients with a history of illicit hormone use and silicone injections. doi:10.1111/tbj.14178

158. Chadwick KA, Coleman R, Andreadis K, Pitti M, Rameau A. Outcomes of Gender-Affirming Voice and Communication Modification for Transgender Individuals. *The Laryngoscope*. 2022;132(8):1615-1621. doi:10.1002/lary.29946

159. Falcone M, Garaffa G, Gillo A, Dente D, Christopher AN, Ralph DJ. Outcomes of inflatable penile prosthesis insertion in 247 patients completing female to male gender reassignment surgery. doi:10.1111/bju.14027

160. Meister J, Kuehn H, Shehata-Dieler W, Kraus F, Hagen R, Kleinsasser N. Patient Satisfaction after Pitch Elevation and Development of a Therapy Algorithm. doi:10.1055/s-0042-103590

161. Weigert R, Frison E, Sessiecq Q, Al Mutairi K, Casoli V. Patient Satisfaction with Breasts and Psychosocial, Sexual, and Physical Well-Being after Breast Augmentation in Male-to-Female Transsexuals. doi:10.1097/01.prs.0000434415.70711.49

162. Davies A, Bouman WP, Richards C, et al. Patient satisfaction with gender identity clinic services in the United Kingdom. doi:10.1080/14681994.2013.834321

163. Bockting W, Miner MH, Swinburne Romine RE, et al. The Transgender Identity Survey: A Measure of Internalized Transphobia. *LGBT Health*. 2020;7(1):15-27. doi:10.1089/lgbt.2018.0265

164. Bockting W, Robinson B, Benner A, Scheltema K. Patient satisfaction with transgender health services (vol 30, pg 277, 2004). doi:10.1080/00926230490507283

165. Lawrence AA. Patient-reported complications and functional outcomes of male-to-female sex reassignment surgery. doi:10.1007/s10508-006-9104-9

166. Bouman MB, van der Sluis WB, Hamstra LEV, et al. Patient-Reported Esthetic and Functional Outcomes of Primary Total Laparoscopic Intestinal Vaginoplasty in Transgender Women With Penoscrotal Hypoplasia. doi:10.1016/j.jsxm.2016.06.009

© 2023 Kamran R et al. *JAMA Network Open.*

167. de Rooij FPW, van de Grift TC, Veerman H, et al. Patient-Reported Outcomes After Genital Gender-Affirming Surgery With Versus Without Urethral Lengthening in Transgender Men. doi:10.1016/j.jsxm.2021.03.002

168. McNichols CHL, O'Brien-Coon D, Fischer B. Patient-reported satisfaction and quality of life after trans male gender affirming surgery. doi:10.1080/26895269.2020.1775159

169. Basar K, Oz G, Karakaya J. Perceived Discrimination, Social Support, and Quality of Life in Gender Dysphoria. doi:10.1016/j.jsxm.2016.04.071

170. Owen-Smith AA, Sineath C, Sanchez T, et al. Perception of community tolerance and prevalence of depression among transgender persons. doi:10.1080/19359705.2016.1228553

171. Holmberg EB, Oates J, Dacakis G, Grant C. Phonetograms, Aerodynamic Measurements, Self-Evaluations, and Auditory Perceptual Ratings of Male-to-Female Transsexual Voice. doi:10.1016/j.jvoice.2009.02.002

172. Silva ED, Fighera TM, Allgayer RM, Lobato MIR, Spritzer PM. Physical and Sociodemographic Features Associated With Quality of Life Among Transgender Women and Men Using Gender-Affirming Hormone Therapy. doi:10.3389/fpsyt.2021.621075

173. Ganor O, Taghinia AH, Diamond DA, Boskey ER. Piloting a Genital Affirmation Surgical Priorities Scale for Trans Masculine Patients. doi:10.1089/trgh.2019.0038

174. Wagner I, Fugain C, Monneron-Girard L, Cordier B, Chabolle F. Pitch-raising surgery in fourteen male-to-female transsexuals. doi:10.1097/00005537-200307000-00011

175. de Vries ALC, Steensma TD, Cohen-Kettenis PT, VanderLaan DP, Zucker KJ. Poor peer relations predict parent- and self-reported behavioral and emotional problems of adolescents with gender dysphoria: a cross-national, cross-clinic comparative analysis. *European Child and Adolescent Psychiatry*. 2016;25(6):579-588. doi:10.1007/s00787-015-0764-7

176. Matthys I, Defreyne J, Elaut E, et al. Positive and Negative Affect Changes during Gender-Affirming Hormonal Treatment: Results from the European Network for the Investigation of Gender Incongruence (ENIGI). doi:10.3390/jcm10020296

177. Sadoughi W, Jayaram B, Bush I. Postoperative changes in the self concept of transsexuals as measured by the Tennessee Self Concept Scale. *Archives of Sexual Behavior*. 1978;7(4):347-349. doi:10.1007/BF01542043

178. Massie JP, Morrison SD, Van Maasdam J, Satterwhite T. Predictors of Patient Satisfaction and Postoperative Complications in Penile Inversion Vaginoplasty. doi:10.1097/prs.0000000000004427

© 2023 Kamran R et al. *JAMA Network Open.*

179. Bouman WP, Davey A, Meyer C, Witcomb GL, Arcelus J. Predictors of psychological well-being among treatment seeking transgender individuals. doi:10.1080/14681994.2016.1184754

180. Elfering L, van der Sluis WB, Bouman MB, et al. Preexpansion in Phalloplasty Patients Is It Effective? doi:10.1097/sap.0000000000001968

181. Kumchenko S, Rasskazova E, Tkhostov A, Emelin V. Preference for Masculine or Feminine Gender Roles and Its Relationship to Well-Being in Transgender Persons: Comparing Pre-Treatment, Hormonal Therapy, and Post-Surgery Groups. doi:10.3390/bs10060100

182. Axfors C, Iliadis SI, Rasmusson LL, et al. Preferences for Gender Affirming Treatment and Associated Factors Among Transgender People in Sweden. doi:10.1007/s13178-021-00650-2

183. Lane M, Kirsch MJ, Sluiter EC, et al. Prevalence of Psychosocial Distress in Transmen Seeking Gender-Affirming Mastectomy. doi:10.1097/prs.0000000000007357

184. Morrison SD, Capitan-Canadas F, Sanchez-Garcia A, et al. Prospective Quality-of-Life Outcomes after Facial Feminization Surgery: An International Multicenter Study. doi:10.1097/prs.0000000000006837

185. Karpel L, Gardel B, Revol M, Bremont-Weil C, Ayoubi JM, Cordier B. Psychological and sexual well being of 207 transsexuals after sex reassignment in France. doi:10.1016/j.amp.2012.01.021

186. de Graaf NM, Cohen-Kettenis PT, Carmichael P, et al. Psychological functioning in adolescents referred to specialist gender identity clinics across Europe: a clinical comparison study between four clinics. *European Child and Adolescent Psychiatry*. 2018;27(7):909-919. doi:10.1007/s00787-017-1098-4

187. Costa R, Dunsford M, Skagerberg E, Holt V, Carmichael P, Colizzi M. Psychological Support, Puberty Suppression, and Psychosocial Functioning in Adolescents with Gender Dysphoria. *J Sex Med 2015 Nov;12(11):2206-14 doi: 10 1111/jsm 13034 Epub 2015 Nov 9*. (11):2206-2214.

188. Shirdel-Havar E, Steensma TD, Cohen-Kettenis PT, Kreukels BPC. Psychological symptoms and body image in individuals with gender dysphoria: A comparison between Iranian and Dutch clinics. doi:10.1080/15532739.2018.1444529

189. Jokic-Begic N, Korajlija AL, Jurin T. Psychosocial Adjustment to Sex Reassignment Surgery: A Qualitative Examination and Personal Experiences of Six Transsexual Persons in Croatia. doi:10.1155/2014/960745

190. Levitan, N., Barkmann, C., Richter-Appelt, H. *et al.* Risk factors for psychological functioning in German adolescents with gender dysphoria: poor peer relations and general family functioning. *Eur Child Adolesc Psychiatry* **28**, 1487–1498 (2019). https://doi.org/10.1007/s00787-019-01308-6


© 2023 Kamran R et al. *JAMA Network Open.*

191. Becker-Hebly I, Fahrenkrug S, Campion F, Richter-Appelt H, Schulte-Markwort M, Barkmann C. Psychosocial health in adolescents and young adults with gender dysphoria before and after gender-affirming medical interventions: a descriptive study from the Hamburg Gender Identity Service. *Eur Child Adolesc Psychiatry*. 2021;30(11):1755-1767. doi:10.1007/s00787-020-01640-2

192. Breidenstein A, Hess J, Hadaschik B, Teufel M, Tagay S. Psychosocial Resources and Quality of Life in Transgender Women following Gender-Affirming Surgery. doi:10.1016/j.jsxm.2019.08.007

193. de Vries AL, Steensma TD, Doreleijers TA, Cohen-Kettenis PT. Puberty suppression in adolescents with gender identity disorder: a prospective follow-up study. *J Sex Med 2011 Aug;8(8):2276-83 doi: 10 1111/j 1743-6109 2010 01943 x Epub 2010 Jul 14*. (8):2276-2283.

194. Masaoka M, Uchida S, Harima K, Hasegawa T. Quality of Life (Qol) and Other Psychological Index in Female-to-Male Transgender/Transsexuals: 1. A Cross-Sectional Study between Those with, Vs. Without Hormone Therapy, 2. A Longitudinal Study of Spontaneous Changes in Those with Hormone Therapy.

195. Kuhn A, Bodmer C, Stadlmayr W, Kuhn P, Mueller MD, Birkhauser M. Quality of life 15 years after sex reassignment surgery for transsexualism. doi:10.1016/j.fertnstert.2008.08.126

196. Simbar M, Nazarpour S, Mirzababaie M, Emam Hadi MA, Ramezani Tehrani F, Alavi Majd H. Quality of Life and Body Image of Individuals with Gender Dysphoria. *J Sex Marital Ther 2018;44(6):523-532 doi: 10 1080/0092623X 2017 1419392 Epub 2018 Feb 12*. (6):523-532.

197. Castellano E, Crespi C, Dell'Aquila C, et al. Quality of life and hormones after sex reassignment surgery. doi:10.1007/s40618-015-0398-0

198. Papadopulos NA, Lelle JD, Zavlin D, et al. Quality of Life and Patient Satisfaction Following Male-to-Female Sex Reassignment Surgery. doi:10.1016/j.jsxm.2017.01.022

199. Ozceltik G, Hortu I, Itil IM, Yeniel AO. Vaginal approach versus laparoscopy for hysterectomy in transgender men. J Gynecol Obstet Hum Reprod. 2022 Feb;51(2):102286. doi:10.1016/j.jogoh.2021.102286. Epub 2021 Dec 12. PMID: 34910989.

200. Gümüşsoy S, Hortu İ, Dal NA, Dönmez S, Mete Ergenoğlu A. Quality of Life and Perceived Social Support Before and After Sex Reassignment Surgery. *Clinical Nursing Research*. 2022;31(3):481-488. doi:10.1177/10547738211040636

201. Papadopulos NA, Ehrenberger B, Zavlin D, et al. Quality of Life and Satisfaction in Transgender Men After Phalloplasty in a Retrospective Study. doi:10.1097/sap.0000000000002693

202. Wierckx K, Van Caenegem E, Weyers S, et al. Quality of Life and Sexual Health after Sex Reassignment Surgery in Female-to-Male Transsexuals.

© 2023 Kamran R et al. *JAMA Network Open.*

203. Naeimi S, Akhlaghdoust M, Chaichian S, et al. Quality of Life Changes in Iranian Patients Undergoing Female-to-Male Transsexual Surgery: A Prospective Study.

204. Agarwal CA, Scheefer MF, Wright LN, Walzer NK, Rivera A. Quality of life improvement after chest wall masculinization in female-to-male transgender patients: A prospective study using the BREAST-Q and Body Uneasiness Test. doi:10.1016/j.bjps.2018.01.003

205. Lindqvist EK, Sigurjonsson H, Mollermark C, Rinder J, Farnebo F, Lundgren TK. Quality of life improves early after gender reassignment surgery in transgender women. doi:10.1007/s00238-016-1252-0

206. Jellestad L, Jaggi T, Corbisiero S, et al. Quality of Life in Transitioned Trans Persons: A Retrospective Cross-Sectional Cohort Study. doi:10.1155/2018/8684625

207. Ainsworth TA, Spiegel JH. Quality of life of individuals with and without facial feminization surgery or gender reassignment surgery. doi:10.1007/s11136-010-9668-7

208. Valashany BT, Janghorbani M. Quality of life of men and women with gender identity disorder. doi:10.1186/s12955-018-0995-7

209. Chaovanalikit T, Wirairat K, Sriswadpong P. Quality of Life, Self-Esteem, and Depression Among Thai Transgender Women Before and After Male-to-Female Gender Confirmation Surgery: A Prospective Cohort Observational Study. *Sexual Medicine*. 2022;10(4). doi:10.1016/j.esxm.2022.100533

210. Alcon A, Kennedy A, Wang E, et al. Quantifying the Psychosocial Benefits of Masculinizing Mastectomy in Trans Male Patients with Patient-Reported Outcomes: The University of California, San Francisco, Gender Quality of Life Survey. doi:10.1097/prs.0000000000007883

211. Arnoldussen M, Steensma TD, Popma A, van der Miesen AIR, Twisk JWR, de Vries ALC. Re-evaluation of the Dutch approach: are recently referred transgender youth different compared to earlier referrals? doi:10.1007/s00787-019-01394-6

212. Niechajev I. Reduction genioplasty for mandibular prognathism and long chin. doi:10.1007/s10006-020-00853-8

213. Khoosal D, Grover P, Terry T. Satisfaction with a gender realignment service. doi:10.1080/14681994.2010.534448

214. Hess J, Neto RR, Panic L, Rubben H, Senf W. Satisfaction With Male-to-Female Gender Reassignment Surgery. doi:10.3238/arztebl.2014.0795

215. Davey A, Arcelus J, Meyer C, Bouman WP. Self-injury among trans individuals and matched controls: prevalence and associated factors. doi:10.1111/hsc.12239

© 2023 Kamran R et al. *JAMA Network Open.*

216. Bultynck C, Pas C, Defreyne J, Cosyns M, den Heijer M, T'Sjoen G. Self-perception of voice in transgender persons during cross-sex hormone therapy. doi:10.1002/lary.26716

217. Cohen-Kettenis PT, van Goozen SH. Sex reassignment of adolescent transsexuals: a follow-up study. *J Am Acad Child Adolesc Psychiatry*. 1997;36(2):263-271.

218. Kuiper B, Cohen-Kettenis P. Sex reassignment surgery: a study of 141 Dutch transsexuals. *Arch Sex Behav*. 1988;17(5):439-457.

219. Smith YLS, Van Goozen SHM, Kuiper AJ, Cohen-Kettenis PT. Sex reassignment: outcomes and predictors of treatment for adolescent and adult transsexuals. doi:10.1017/s0033291704002776

220. De Cuypere G, T'Sjoen G, Beerten R, et al. Sexual and physical health after sex reassignment surgery. doi:10.1007/s10508-005-7926-5

221. Wierckx K, Elaut E, Van Hoorde B, et al. Sexual Desire in Trans Persons: Associations with Sex Reassignment Treatment. doi:10.1111/jsm.12365

222. Monteiro Petry Jardim LM, Cerentini TM, Lobato MIR, et al. Sexual Function and Quality of Life in Brazilian Transgender Women Following Gender-Affirming Surgery: A Cross-Sectional Study. *International Journal of Environmental Research and Public Health*. 2022;19(23). doi:10.3390/ijerph192315773

223. Hensle TW, Shabsigh A, Shabsigh R, Reiley EA, Meyer-Bahlburg HFL. Sexual Function Following Bowel Vaginoplasty. *Journal of Urology*. 2006;175(6):2283-2286. doi:10.1016/S0022-5347%2806%2900337-5

224. Sahin S, Korkmaz OP, Durcan E, Ozkaya HM, Turan S, Kadioglu P. Sexual Functions of Transgender Individuals Before Gender Transition. doi:10.25179/tjem.2021-81979

225. Badolucci C, Gomez-Gil E, Salamero M, et al. Sexual Quality of Life in Gender-Dysphoric Adults before Genital Sex Reassignment Surgery. doi:10.1111/jsm.12758

226. Hess J, Henkel A, Bohr J, et al. Sexuality after Male-to-Female Gender Affirmation Surgery. *Biomed Res Int 2018 May 27;2018:9037979 doi: 10 1155/2018/9037979 eCollection 2018*.:9037979.

227. Bowen AE, Staggs S, Kaar J, Nokoff N, Simon SL. Short sleep, insomnia symptoms, and evening chronotype are correlated with poorer mood and quality of life in adolescent transgender males. doi:10.1016/j.sleh.2021.03.008

228. Skewis LF, Bretherton I, Leemaqz SY, Zajac JD, Cheung AS. Short-Term Effects of Gender-Affirming Hormone Therapy on Dysphoria and Quality of Life in Transgender Individuals: A Prospective Controlled Study. doi:10.3389/fendo.2021.717766


© 2023 Kamran R et al. *JAMA Network Open.*

229. Hage JJ, Bouman FG. Silicone genital prosthesis for female-to-male transsexuals. *Plast Reconstr Surg*. 1992;90(3):516-519.

230. Butler RM, Horenstein A, Gitlin M, et al. Social Anxiety Among Transgender and Gender Nonconforming Individuals: The Role of Gender-Affirming Medical Interventions. doi:10.1037/abn0000399

231. Fisher AD, Bandini E, Casale H, et al. Sociodemographic and Clinical Features of Gender Identity Disorder: An Italian Multicentric Evaluation. doi:10.1111/j.1743-6109.2012.03006.x

232. Bertrand B, Perchenet AS, Philandrianos C, Casanova D, Cristofari S. Subcutaneous Mastectomy Improves Satisfaction with Body and Psychosocial Function in Trans Men: Findings of a Cross-Sectional Study Using the BODY-Q Chest Module. *Plast Reconstr Surg*. 2019;144(1):144e-145e. doi:10.1097/PRS.0000000000005734

233. van de Grift TC, Elfering L, Greijdanus M, et al. Subcutaneous Mastectomy Improves Satisfaction with Body and Psychosocial Function in Trans Men: Findings of a Cross-Sectional Study Using the BODY-Q Chest Module. doi:10.1097/prs.0000000000004827

234. Marco F, Massimiliano T, Marco O, et al. Suprapubic pedicled phalloplasty in transgender men: a multicentric retrospective cohort analysis. doi:10.1038/s41443-020-0238-4

235. Stein MJ, Grigor E, Hardy J, Jarmuske M. Surgical and patient-reported outcomes following double incision and free nipple grafting for female to male gender affirmation: does obesity make a difference? doi:10.1016/j.bjps.2020.12.004

236. van de Grift TC, Elfering L, Bouman MB, Buncamper ME, Mullender MG. Surgical Indications and Outcomes of Mastectomy in Transmen: A Prospective Study of Technical and Self-Reported Measures. doi:10.1097/prs.0000000000003607

237. Terrier J, Courtois F, Ruffion A, Morel Journel N. Surgical outcomes and patients' satisfaction with suprapubic phalloplasty. *J Sex Med 2014 Jan;11(1):288-98 doi: 10 1111/jsm 12297 Epub 2013 Sep 12*. (1):288-298.

238. Ascha M, Massie JP, Morrison SD, Crane CN, Chen ML. Surgical Outcomes Following Gender Affirming Penile Reconstruction: Patient-Reported Outcomes From a Multi-Center, International Survey of 129 Transmasculine Patients. *J Urol 2018 Jan;199(1):206-214 doi: 10 1016/j juro 2017 07 084 Epub 2017 Jul 29*. (4):800-811.

239. Amend B, Seibold J, Toomey P, Stenzl A, Sievert KD. Surgical Reconstruction for Male-to-Female Sex Reassignment. doi:10.1016/j.eururo.2012.12.030

240. van de Grift TC, Elaut E, Cerwenka SC, Cohen-Kettenis PT, Kreukels BPC. Surgical Satisfaction, Quality of Life, and Their Association After Gender-Affirming Surgery: A Follow-up Study. doi:10.1080/0092623x.2017.1326190

© 2023 Kamran R et al. *JAMA Network Open.*

241. Grannis C, Leibowitz SF, Gahn S, et al. Testosterone treatment, internalizing symptoms, and body image dissatisfaction in transgender boys. doi:10.1016/j.psyneuen.2021.105358

242. Gava G, Mancini I, Cerpolini S, Baldassarre M, Seracchioli R, Meriggiola MC. Testosterone undecanoate and testosterone enanthate injections are both effective and safe in transmen over 5 years of administration. doi:10.1111/cen.13821

243. Fleming MZ, MacGowan BR, Robinson L, Spitz J, Salt P. The body image of the postoperative female-to-male transsexual. *Journal of Consulting and Clinical Psychology*. 1982;50(3):461-462. doi:10.1037/0022-006X.50.3.461

244. Carew L, Dacakis G, Oates J. The effectiveness of oral resonance therapy on the perception of femininity of voice in male-to-female transsexuals. doi:10.1016/j.jvoice.2006.05.005

245. Bettocchi C, Palumbo F, Cormio L, Ditonno P, Battaglia M, Selvaggi FP. The effects of androgen depletion on human erectile function: a prospective study in male-to-female transsexuals. doi:10.1038/sj.ijir.3901216

246. Jones BA, Bouman WP, Haycraft E, Arcelus J. The Gender Congruence and Life Satisfaction Scale (GCLS): Development and validation of a scale to measure outcomes from transgender health services. doi:10.1080/15532739.2018.1453425

247. McGuire JK, Beek TF, Catalpa JM, Steensma TD. The Genderqueer Identity (GQI) Scale: Measurement and validation of four distinct subscales with trans and LGBQ clinical and community samples in two countries. doi:10.1080/15532739.2018.1460735

248. Asadi M, Tabari F, Haghani S, Heidari ME. The impact of empowerment model-based education on quality of life of transgender people under hormone therapy: A randomized clinical trial. doi:10.4103/jfmpc.jfmpc_1201_19

249. Nuyen B, Kandathil C, McDonald D, Thomas J, Most SP. The impact of living with transfeminine vocal gender dysphoria: Health utility outcomes assessment. doi:10.1080/26895269.2021.1919277

250. Spanos C, Grace JA, Leemaqz SY, et al. The Informed Consent Model of Care for Accessing Gender-Affirming Hormone Therapy Is Associated With High Patient Satisfaction. doi:10.1016/j.jsxm.2020.10.020

251. Bustos SS, Forte AJ, Ciudad P, Manrique OJ. The Nipple Split Sharing vs. Conventional Nipple Graft Technique in Chest Wall Masculinization Surgery: Can We Improve Patient Satisfaction and Aesthetic Outcomes? doi:10.1007/s00266-020-01803-1

252. Rakic Z, Starcevic V, Maric J, Kelin K. The outcome of sex reassignment surgery in Belgrade: 32 patients of both sexes. doi:10.1007/bf02437545

© 2023 Kamran R et al. *JAMA Network Open.*

253. Rehman J, Lazer S, Benet AE, Schaefer LC, Melman A. The reported sex and surgery satisfactions of 28 postoperative male-to-female transsexual patients. doi:10.1023/a:1018745706354

254. Vukadinovic V, Stojanovic B, Majstorovic M, Milosevic A. The Role of Clitoral Anatomy in Female to Male Sex Reassignment Surgery. doi:10.1155/2014/437378

255. Glynn TR, Gamarel KE, Kahler CW, Iwamoto M, Operario D, Nemoto T. The role of gender affirmation in psychological well-being among transgender women. *Psychology of Sexual Orientation and Gender Diversity*. 2016;3(3):336-344. doi:10.1037/sgd0000171

256. Noureai SAR, Randhawa P, Andrews PJ, Saleh HA. The role of nasal feminization rhinoplasty in male-to-female gender reassignment. doi:10.1001/archfaci.9.5.318

257. Thammapiwan P, Suwan A, Panyakhamlerd K, Suwajo P, Phanuphak N, Taechakraichana N. The sexual function among transgender women who have undergone gender-affirming surgery using penile skin inversion vaginoplasty in Thailand. doi:10.1007/s00238-021-01857-7

258. Al-Tamimi M, Pigot GL, van der Sluis WB, et al. The Surgical Techniques and Outcomes of Secondary Phalloplasty After Metoidioplasty in Transgender Men: An International, Multi-Center Case Series. doi:10.1016/j.jsxm.2019.07.027

259. Pauli D, Gunthardt M, Schenker T, et al. The Zurich Specialist Clinic for Adolescent with Gender Dysphoria - Preliminary Follow-up Results. doi:10.13109/prkk.2020.69.6.570

260. Ascha M, Sasson DC, Sood R, et al. Top Surgery and Chest Dysphoria Among Transmasculine and Nonbinary Adolescents and Young Adults. *Jama Pediatrics*. 2022;176(11):1115-1122. doi:10.1001/jamapediatrics.2022.3424

261. Holt NR, Huit TZ, Shulman GP, et al. Trans Collaborations Clinical Check-In (TC3): Initial Validation of a Clinical Measure for Transgender and Gender Diverse Adults Receiving Psychological Services. doi:10.1016/j.beth.2019.04.001

262. Hancock AB, Childs KD, Irwig MS. Trans Male Voice in the First Year of Testosterone Therapy: Make No Assumptions. doi:10.1044/2017_jslhr-s-16-0320

263. Ho F, Mussap AJ. Transgender Mental Health in Australia: Satisfaction with Practitioners and the Standards of Care. doi:10.1111/ap.12188

264. Nelson L, Whallett EJ, McGregor JC. Transgender patient satisfaction following reduction mammaplasty. doi:10.1016/j.bjps.2007.10.049

265. Parra N, Patete C, Tarsha A, Fein LA. Transgender patients’ satisfaction with their mental health providers prior to gender affirming surgery. doi:10.1080/19359705.2020.1793868

© 2023 Kamran R et al. *JAMA Network Open.*

266. Hobson BJ, Lett E, Hawkins LA, Swendiman RA, Nance ML, Dowshen NL. Transgender Youth Experiences with Implantable GnRH Agonists for Puberty Suppression. doi:10.1089/trgh.2021.0006

267. Morsomme D, Revis J, Thomas E. Translation, Adaptation, and Preliminary Validation of Dacakis and Davies' "Transsexual Voice Questionnaire (Male to Female)" in French. doi:10.1016/j.jvoice.2018.03.001

268. Mora E, Carrillo A, Giribet A, Becerra A, Lucio MJ, Cobeta I. Translation, Cultural Adaptation, and Preliminary Evaluation of the Spanish Version of the Transgender Voice Questionnaire for Male-to-Female Transsexuals (TVQ(MtF)). doi:10.1016/j.jvoice.2017.05.012

269. van de Grift TC, Pigot GLS, Kreukels BPC, Bouman MB, Mullender MG. Transmen's Experienced Sexuality and Genital Gender-Affirming Surgery: Findings From a Clinical Follow-Up Study. doi:10.1080/0092623x.2018.1500405

270. Colizzi M, Costa R, Todarello O. Transsexual patients' psychiatric comorbidity and positive effect of cross-sex hormonal treatment on mental health: Results from a longitudinal study. doi:10.1016/j.psyneuen.2013.09.029

271. Schwarz K, Fontanari AMV, Mueller A, et al. Transsexual Voice Questionnaire for Male-to-female Brazilian Transsexual People. doi:10.1016/j.jvoice.2016.02.012

272. Bodlund O, Kullgren G. Transsexualism - General outcome and prognostic factors: A five-year follow-up study of nineteen transsexuals in the process of changing sex. doi:10.1007/bf02438167

273. Hunt D, Hampson JL. Transsexualism: A standardized psychosocial rating format for the evaluation of results of sex reassignment surgery. *Archives of Sexual Behavior*. 1980;9(3):255-263. doi:10.1007/BF01542251

274. Zimmermann A, Zimmer R, Kovacs L, et al. Transsexuals' life satisfaction after gender transformation operations. doi:10.1007/s00104-005-1138-3

275. Hepp U, Klaghofer R, Burkhard-Kubler R, Buddeberg C. Treatment history of transsexual patients: a retrospective follow-up study. doi:10.1007/s00115-001-1225-8

276. Garcia MM, Shen W, Zhu R, et al. Use of right colon vaginoplasty in gender affirming surgery: proposed advantages, review of technique, and outcomes. doi:10.1007/s00464-020-08078-2

277. Rachlin K, Green J, Lombardi E. Utilization of health care among female-to-male transgender individuals in the united states. doi:10.1080/00918360801982124

278. Ozkan O, Cinpolat A, Dogan NU, et al. Vaginal reconstruction with the modified rectosigmoid colon: surgical technique, long-term results and sexual outcomes. doi:10.1080/2000656x.2018.1444616

© 2023 Kamran R et al. *JAMA Network Open.*

279. Salm S, Hower K, Neumann S, Ansmann L. Validation of the German Version of the Transsexual Voice Questionnaire for Male-to-Female Transsexuals. doi:10.1016/j.jvoice.2018.06.010

280. To M, Zhang Q, Bradlyn A, et al. Visual Conformity With Affirmed Gender or "Passing": Its Distribution and Association With Depression and Anxiety in a Cohort of Transgender People. doi:10.1016/j.jsxm.2020.07.019

281. Young VN, Yousef A, Zhao NW, Schneider SL. Voice and Stroboscopic Characteristics in Transgender Patients Seeking Gender-Affirming Voice Care. *Laryngoscope 2021 May;131(5):1071-1077 doi: 10 1002/lary 28932 Epub 2020 Aug 4*. (5):1071-1077.

282. Casado JC, Rodriguez-Parra MJ, Adrian JA. Voice feminization in male-to-female transgendered clients after Wendler's glottoplasty with vs. without voice therapy support. doi:10.1007/s00405-016-4420-8

283. Hancock AB, Krissinger J, Owen K. Voice Perceptions and Quality of Life of Transgender People. doi:10.1016/j.jvoice.2010.07.013

284. Casado JC, Connor C, Angulo MS, Adrian JA. Wendler glottoplasty and voice-therapy in male-to-female transsexuals: results in pre and post-surgery assessment. doi:10.1016/j.otorri.2015.02.003

285. Cardoso da Silva D, Schwarz K, Fontanari AM, et al. WHOQOL-100 Before and After Sex Reassignment Surgery in Brazilian Male-to-Female Transsexual Individuals. *J Sex Med 2016 Jun;13(6):988-93 doi: 10 1016/j jsxm 2016 03 370 Epub 2016 Apr 21*. (6):988-993.

286. de Vries AL, McGuire JK, Steensma TD, Wagenaar EC, Doreleijers TA, Cohen-Kettenis PT. Young adult psychological outcome after puberty suppression and gender reassignment. *Pediatrics 2014 Oct;134(4):696-704 doi: 10 1542/peds 2013-2958 Epub 2014 Sep 8*. (4):696-704.

© 2023 Kamran R et al. *JAMA Network Open.*
