# Supplementary material for: Implementation of Patient-Reported Outcome Measures for Gender-Affirming Care Worldwide: A Systematic Review
Source: JAMA Netw Open. 2023 Apr 3;6(4):e236425. doi: 10.1001/jamanetworkopen.2023.6425 (PMC10071345; doi:10.1001/jamanetworkopen.2023.6425)
Supplement: Supplement 2. — Data Sharing Statement [file jamanetwopen-e236425-s002.pdf]

Data Sharing Statement

Kamran. Implementation of Patient-Reported Outcome Measures for Gender-Affirming Care Worldwide. *JAMA Netw Open*. Published April 03, 2023.
doi:10.1001/jamanetworkopen.2023.6425

Data
Data available: No

Additional Information
Explanation for why data not available: Please contact the authors if you would like to review data from this study
